# Characterization of substantia nigra neurogenesis in homeostasis and dopaminergic degeneration: beneficial effects of the microneurotrophin BNN-20

**DOI:** 10.1186/s13287-021-02398-3

**Published:** 2021-06-10

**Authors:** Theodora Mourtzi, Dimitrios Dimitrakopoulos, Dimitrios Kakogiannis, Charalampos Salodimitris, Konstantinos Botsakis, Danai Kassandra Meri, Maria Anesti, Aggeliki Dimopoulou, Ioannis Charalampopoulos, Achilleas Gravanis, Nikolaos Matsokis, Fevronia Angelatou, Ilias Kazanis

**Affiliations:** 1 Department of Physiology, Medical School, University of Patras, 26504 Patras, Greece; 2 Lab of Developmental Biology, Department of Biology, University of Patras, 26500 Patras, Greece; 3 Lab of Human and Animal Physiology, Department of Biology, University of Patras, 26500 Patras, Greece; 4 Department of Pharmacology, Medical School, University of Crete, 71500 Heraklion, Greece; 5 Institute of Molecular Biology and Biotechnology, Foundation for Research and Technology-Hellas, 70013 Heraklion, Greece

**Keywords:** Neurogenesis; Adult brain, Dopaminergic, Substantia nigra, Parkinson’s disease, Weaver mouse, Microneurotrophin, BNN-20, Neurotrophic, Subependymal zone

## Abstract

**Background:**

Loss of dopaminergic neurons in the substantia nigra pars compacta (SNpc) underlines much of the pathology of Parkinson’s disease (PD), but the existence of an endogenous neurogenic system that could be targeted as a therapeutic strategy has been controversial. BNN-20 is a synthetic, BDNF-mimicking, microneurotrophin that we previously showed to exhibit a pleiotropic neuroprotective effect on the dopaminergic neurons of the SNpc in the “weaver” mouse model of PD. Here, we assessed its potential effects on neurogenesis.

**Methods:**

We quantified total numbers of dopaminergic neurons in the SNpc of wild-type and “weaver” mice, with or without administration of BNN-20, and we employed BrdU labelling and intracerebroventricular injections of DiI to evaluate the existence of dopaminergic neurogenesis in the SNpc and to assess the origin of newborn dopaminergic neurons. The in vivo experiments were complemented by in vitro proliferation/differentiation assays of adult neural stem cells (NSCs) isolated from the substantia nigra and the subependymal zone (SEZ) stem cell niche to further characterize the effects of BNN-20.

**Results:**

Our analysis revealed the existence of a low-rate turnover of dopaminergic neurons in the normal SNpc and showed, using three independent lines of experiments (stereologic cell counts, BrdU and DiI tracing), that the administration of BNN-20 leads to increased neurogenesis in the SNpc and to partial reversal of dopaminergic cell loss. The newly born dopaminergic neurons, that are partially originated from the SEZ, follow the typical nigral maturation pathway, expressing the transcription factor FoxA2. Importantly, the pro-cytogenic effects of BNN-20 were very strong in the SNpc, but were absent in other brain areas such as the cortex or the stem cell niche of the hippocampus. Moreover, although the in vitro assays showed that BNN-20 enhances the differentiation of NSCs towards glia and neurons, its in vivo administration stimulated only neurogenesis.

**Conclusions:**

Our results demonstrate the existence of a neurogenic system in the SNpc that can be manipulated in order to regenerate the depleted dopaminergic cell population in the “weaver” PD mouse model. Microneurotrophin BNN-20 emerges as an excellent candidate for future PD cell replacement therapies, due to its area-specific, pro-neurogenic effects.

**Supplementary Information:**

The online version contains supplementary material available at 10.1186/s13287-021-02398-3.

## Background

Parkinson’s disease (PD) is the second most common neurodegenerative disease, constituting a significant clinical and socioeconomic problem [1]. No established therapy to slow down, stop, or reverse the degenerative process exists and symptomatic treatments hold significant side effects [2, 3], although recent experimental work has showed that the nigrostriatal pathway can be restored by grafting [4] or reprogramming cells [5, 6]. Neurotrophic factors, such as brain-derived neurotrophic factor (BDNF) and glial-derived neurotrophic factor (GDNF), are well-described candidates for the treatment of PD, as they exhibit dual neuroprotective and neurogenic properties. However, their clinical use is hampered by the limited penetration of the blood-brain barrier (BBB), due to their large molecular size and their poor pharmacokinetic properties [2, 7–9].

Research on the pharmacotherapy of PD has recently focused on micromolecular compounds that can mimic the neuroprotective and neurogenic properties of endogenous neurotrophic factors, while penetrating the BBB. Microneurotrophins (MNTs) are micromolecular synthetic analogues of dehydroepiandrosterone (DHEA) that selectively bind and activate the neurotrophin receptors TrkA (of NGF), TrkB (of BDNF), and p75^NTR^, mimicking the beneficial effects of growth factors, which can also penetrate the BBB. Furthermore, MNTs are not activating classical estrogen or androgen receptors and, hence, are considered to be deprived of hormone-dependent side effects [10–13].

The “weaver” mouse model is the only genetic model of progressive nigrostriatal dopaminergic neurodegeneration, caused by an autosomal recessive point mutation of the Girk2 potassium channel, with dopaminergic cell loss in the substantia nigra pars compacta (SNpc) starting on postnatal day (P) 7 and reaching the 75% of cells on P60. The mutation leads to the loss of Girk2’s ion specificity, and to the progressive loss of the dopaminergic neurons of the SNpc and other brain regions, due to excitotoxicity. Even though Girk2 mutations have not been identified in human cases of PD so far, the model exhibits many significant hallmarks of the disease, such as neuroinflammation [13–15], oxidative stress [13, 16, 17], dopamine reduction in the striatum [15, 18], motor deficits [15, 18, 19], a-synuclein pathology [17], reduced levels of BDNF [13, 15], and cognitive impairment in the more progressed stages of degeneration [19]. Hence, it consists a phenocopy of human PD ideal for neuroprotection studies.

We have recently reported the strong neuroprotective properties (antioxidant, antiapoptotic, anti-inflammatory) of the MNT BNN-20 (17β-spiro-(androst-5-en-17,2'-oxiran)-3β-ol) in the “weaver” mouse model [13, 15]. Furthermore, we have shown that BNN-20 exerts its beneficial effects, at least in part, by activating the TrkB receptors and their downstream signaling pathways TrkB-Akt-NFκΒ and TrkB-ERK1/2-NFκΒ, triggering an NFκΒ-dependent positive feedback loop, which leads to the increase of BDNF itself [13, 15].

During the last two decades, the existence of neurogenesis in noncanonical sites of the adult rodent brain has been increasingly supported by several research groups [20, 21]. However, the occurrence of adult dopaminergic neurogenesis in the SNpc remains controversial until now [22–28]. Here, we use exhaustive dopaminergic neuron cell counts and we employ BrdU and DiI cell tracing analyses, in order to investigate the existence of endogenous dopaminergic neurogenesis in the SNpc of postnatal wild-type and “weaver” mice, as well as the possible beneficial effect of BNN-20 on it. Our findings demonstrate that new dopaminergic neurons are generated in the normal and the degenerated SNpc and that this system can be exogenously manipulated. Administration of BNN-20 led to the specific increase in dopaminergic SNpc neurogenesis, without affecting gliogenesis and the hippocampal neural stem cell niche. These, along with our recently published results, make BNN-20 a strong candidate molecule for the treatment of PD.

## Methods

### Animal maintenance and handling

All experiments were performed with male and female homozygous “weaver” (Aw-J/A-Kcnj6wv/J) mice of the B6CBAC strain and age-matched B6CBAC wild-type animals. The breeding and handling of the animals was performed in accordance to the European Communities Council Directive Guidelines (86/609/EEC) for the care and use of Laboratory animals as implemented in Greece by the Presidential Decree 56/2013 and approved and scrutinized by the Prefectural Animal Care and Use Committee (No: EL 13BIO04) and the Animal Welfare and Ethical Review Committee of the University of Patras. Animals were maintained in steady light/dark cycle (12/12 h) with free access to food and water. Identification of homozygotes (here called “weaver” or wv) was performed based on behavior and randomly confirmed by PCR. “Weaver” phenotype involved weakness, hypotonia, extensive periods of resting and movement-initiated tremor, poor limb coordination, and instability of gait (due to their underdeveloped cerebellum) [29]. “Weaver” mice were fed daily with a paste consisting of standard rodent food pellets and water, as they are unable to reach the water bottles, due to their limited mobility. They are smaller in size and have increased morbidity, especially before weaning.

BNN-20 (1 mg/ml in 1% ethanol, 0,9% NaCl) (Bionature E.A. Ltd, Nicosia, Cyprus) or vehicle was administered via i.p. injection daily (100 mg BNN-20 per kg b.w.), during P14–P40 or P14–P60. For the labelling of proliferating cells, bromodeoxyuridine (BrdU) was dissolved in phosphate-buffered saline (PBS) (1 mg/ml) and administered daily via i.p. injections (100 mg/kg b.w.), from P20 to P40 or from P40-P60 (Additional Figure 1).

For immunofluorescence analyses, mice were killed on P40 or P60 by intracardial perfusion of 4% paraformaldehyde. Brain tissue was cryoprotected and sliced using a Bright Cryostat (Leica, CM1500) in sections of 15 μm. For neural stem cell (NSC) cultures, mice were killed by cervical dislocation and the SEZ, or the area containing the substantia nigra (referred from now on as SN), were dissected under the stereoscope using anatomical landmarks.

### NSC cultures

The SEZ, or the broader midbrain area of the substantia nigra (carefully excluding all of the periventricular areas) (SN), were dissected under a stereoscope, were dissociated in accutase (37^o^C, 15 min; BIOWEST), and were resuspended in NSC proliferation medium [Dulbecco’s modified Eagle’s medium − high glucose + pyruvate (Thermo Fisher Scientific 11965-084), 20 ng/ml FGF-2 (Peprotech), 20 ng/ml EGF (Peprotech), and 2% B27 (Gibco, 17-504-044)]. NSCs grew as 3D free-floating aggregates called neurospheres that could be passaged. In proliferation or differentiation assays, dissociated neurosphere cells were plated on glass coverslips, coated with poly-d-lysine (PDL), in the presence of either the standard NSC medium (proliferation assays), or after withdrawal of growth factors [in DMEM, 2% B27, 1% N2 (Thermo Fisher)], respectively. Cells were kept for 5 days for proliferation (5dp) and 3, 5, or 7 days (3dd, 5dd, 7dd) for differentiation. BNN-20 (100 nM) was added in the medium for the total culture duration. Cells were fixed with 2% PFA and immunostained. In cell proliferation/differentiation assays, cell counts were performed in at least 5 random optical fields per coverslip per experiment.

In the primary neurosphere assays, spheres were spun down and plated on PDL-coated coverslips, allowed to adhere and subsequently fixed and immunostained. Neurosphere size was calculated by measuring their diameter using ImageJ. In the spontaneous neurogenesis assessment experiment, primary spheres were grown in proliferation medium and were counted as βIII tubulin+ if they included even one βIII tubulin+ cell.

### Dopaminergic neuron quantifications in the SNpc

The total number of dopaminergic neurons of the substantia nigra pars compacta (SNpc) was evaluated by DAB immunostaining for the tyrosine hydroxylase (TH) dopaminergic cell marker. Images were obtained with the × 10 objective (Zeiss, Axio Lab.A1). The SNpc was determined based on the stereotaxic coordinates of Frankin & Paxinos [30]. The total TH+ cell number of the SNpc was quantified stereologically: all TH+ cells were counted in both hemispheres in 10 equally distanced sections (100 μm step) in each animal (*n* = 4 per group). The total dopaminergic cell number was determined using the following formula, appropriate for stereological cell quantification [31] as performed previously [13, 15]:

Number of neurons = 1/ssf (slice sample fraction) × 1/asf (area sample fraction) × 1/tsf (thickness sampling fraction) × number of objects counted

### DiI labelling

Neural stem and progenitor cells (NSPCs) of the SEZ were labelled via stereotaxic injection of the lipophilic dye DiI, as has been previously described [25]. Briefly, 2 μl of DiI solution [10% DiI in dimethyl sulfoxide (DMSO), Sigma] was injected unilaterally in the lateral ventricle of P45 mice (*n* = 3 per animal group), at the following coordinates from bregma [30]: anterior 5 mm; left 0.65 mm; depth 2.3 mm. BNN-20 or saline was co-administered during P14–P60, as described in “Animal maintenance and handling”. Mice were killed after 15 days (at P60).

### Immunofluorescence on brain sections

Tissue sections were thawed and post-fixed with 4% paraformaldehyde for 5 min. For nuclear antigens, a step of antigen retrieval was applied (citrate buffer 0.1 M, pH = 6.0; 100^o^C for 15 min). For BrdU detection, an additional step of DNA denaturation was used, by incubating the sections in 2 M HCl for 1 h at 37 °C, followed by pH neutralization using sodium borate buffer (0,1 M, pH 8.5). Sections were blocked in 10% normal donkey serum. Primary antibody incubation was performed overnight at 4 °C. Secondary antibody incubation was performed for 1 h at RT, using appropriate antibodies conjugated with fluorescent dyes (appropriate AlexaFluor Donkey IgG, 568, 488, or 647, Thermo Fisher Scientific). The list of the used primary antibodies and appropriate dilutions used for immunofluorescence for both brain sections and cell cultures is given below: *Ascl1 (MASH-1)* [mouse monoclonal, Clone 24B72D11.1 (RUO), BD Bioscience, 556604 (1:100)]; *BrdU* [rat monoclonal [BU1/75 (ICR1)], Abcam, ab6326 (1:150)]; *doublecortin (Dcx)* [rabbit polyclonal, Abcam, ab18723 (1:500)]; *GFAP* [chicken, Abcam, ab4674 (1:500)]; *GFAP* [goat polyclonal, Abcam, ab53554 (1:500)]; *GFAP* [mouse monoclonal, Merck-Millipore, G6171 (1:500)]; *HNF-3β (FoxA2)* [mouse monoclonal (RY-7), SantaCruz Biotechnologies, sc-101060 (1:200)]; *Ki67* [rabbit polyclonal [SP6], Abcam, ab16667 (1:500)]; *PCNA* [mouse monoclonal [PC10] (ab29) (1:500)]; *phosphohistone 3 (PH3)* [rabbit polyclonal (phospho S10), Abcam, ab5176 (1:500)]; *Sox2* [goat polyclonal (Y-17), SantaCruz Biotechnologies, sc-17320 (1:200)]; *tyrosine hydroxylase (TH)* [rabbit polyclonal, Merck-Millipore, AB152 (1:300)]; *β-ΙΙΙ-tubulin* [mouse monoclonal [TU-20], Abcam, ab7751 (1:500)]. Images were obtained by fluorescence (Zeiss, Axio Observer.D1) or confocal (Leica TCS SP8) microscopy, using the × 40 and × 63 objectives for brain sections and cells, respectively, and were saved in a high-resolution .tiff format. Quantifications were performed using the LasX (Leica), ImageJ, and Adobe Photoshop CS6 software. For the cell-type profile analysis in the SNpc, the SEZ and the cortex images of at least 5 random optical fields were taken from each area of both hemispheres, from at least 3 brain sections per animal. In the absence of TH immunostaining, the SNpc was identified using anatomical landmarks and adjacent sections already stained for TH. After BrdU immunostainings, the nuclear counterstaining was of low quality; therefore, total nuclei were not quantified. For quantifications in the SEZ, optical fields were obtained from at least three distant areas along the dorso-ventral axis (dorsal, middle, and ventral SEZ) and cells were counted within a 30-μm zone from the ventricular surface that includes the niche [32]. Quantifications of BrdU+ cells within the hippocampus were performed by counting total immunopositive cells in at least 3 optical fields per hippocampus in at least 3 sections per animal and the numbers were normalized per length of the SGZ zone.

### PCR for genotyping for the “weaver” (Girk2) mutation

DNA primers sequences used for genotyping are provided below (5′➔3′):

“wild-type”-specific forward primer: GAGACAGAAACCACCATCG

“weaver”-specific forward primer: GAGACAGAAACCACCATCA

Reverse primer (common for both genotypes): CACGGACTGGATTAAGAGGAGAATAAT

The PCR protocol used was the following (27 cycles): (i) pro-incubation (95 °C, 15 min), (ii) denaturation (95 °C, 30 s), (iii) annealing (54.5^ο^C, 30 s), (iv) extension (72 °C, 1 min), (v) final step (72 °C, 10 min).

### Cell counts—analysis of morphology

For morphological analysis of dopaminergic cells, images from sections immunostained for TH and FoxA2 were taken using confocal microscopy with the × 63 objective lens and a further zoom = 2, at 0.10 μm steps (*n* = 10 cells per group from 2 animals). The longest dimension of the cell body was identified and measured (all measurements taken using the LasX software), followed by measurement of the maximum length at the perpendicular axis and the calculation of the depth of the cell body using the number of steps that included each cell (Visual details on the morphological analysis methodology are provided as Additional Material (See Additional File 2)). One-way ANOVA was employed for each dimension, followed by post hoc analysis.

### Statistics

Statistical analysis was performed by Student’s t test, one-way ANOVA, or two-way ANOVA (depending on the experimental design) using IBM SPSS Statistics 25 and Microsoft Excel. In cases of statistically significant differences (*p* < 0.05), ANOVAs were followed by either LSD or Bonferroni post hoc analysis for multiple comparisons between groups.

## Results

### Long-term BNN-20 treatment inhibits and partially reverses further TH+ cell loss in the SNpc of the “weaver” mouse

**Fig. 1:**
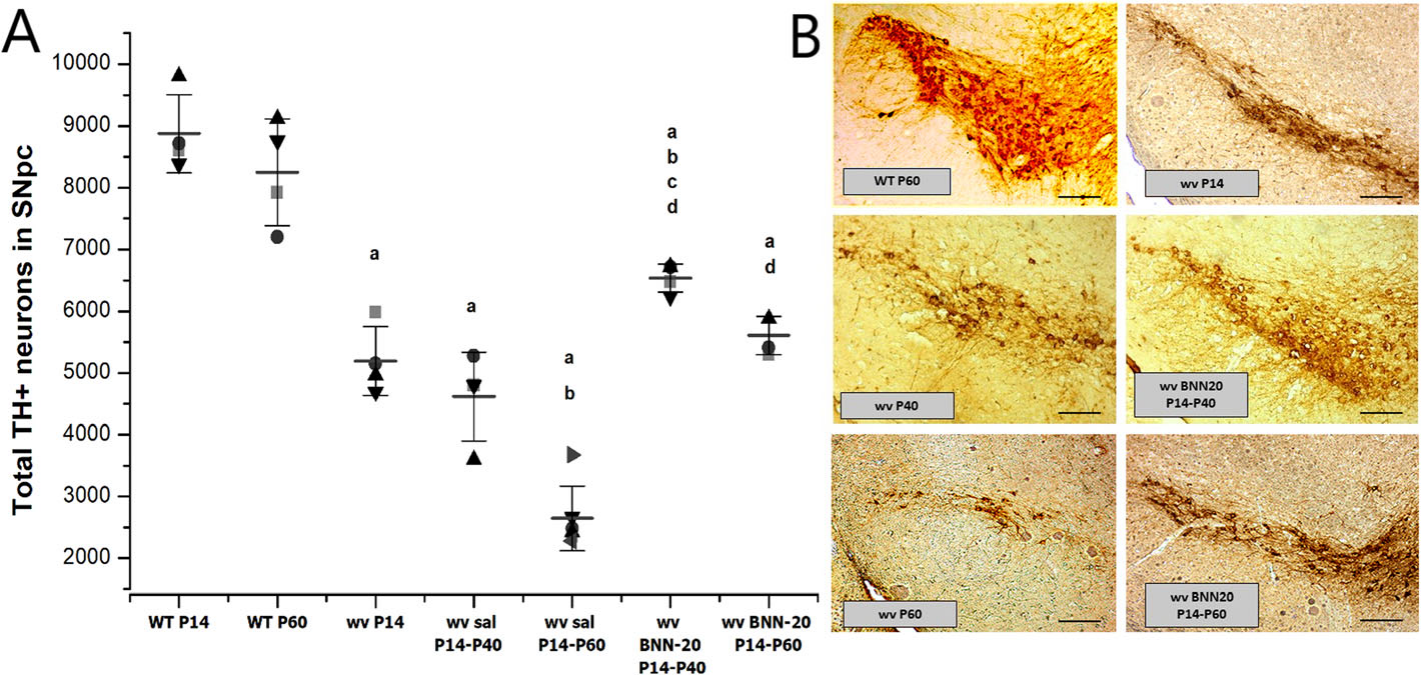
Effects of BNN-20 administration on the total dopaminergic cell number of the “weaver” SNpc. **A** Dot plot of stereologically calculated total TH+ cell numbers in the SNpc of wild-type (WT) and “weaver” (wv) mice, treated with either BNN-20 or saline (sal) from P14 to P40 or to P60 (*n* = 4 per group) [a: *p* < 0.001 compared to WT P14, b: *p* < 0.01 compared to wv P14, c: *p* < 0.001 compared to wv P40, d: *p* < 0.001 compared to wv P60, using two-way ANOVA followed by Bonferroni post hoc test (*p* = 0.000, F = 52.160 for drug, *p* = 0.000, F = 100.234 for genotype and *p* = 0.000, F = 12.114 for the interference of the two variables). Error bars are SDs.]. **B** Representative images of the SNpc using DAB immunohistochemistry against TH [scale bar= 200 μm]

We have previously shown the strong neuroprotective effect of early BNN-20 administration (postnatal days P0-P20, before the initiation and during the first stages of neurodegeneration) on the dopaminergic neurons of the “weaver” (wv) SNpc, including its protective effect on their terminals in the striatum, as well as at a later stage of degeneration (P14–P60), when administration begins after 41% of the dopaminergic neurons are already depleted [13, 15]. Our next goal was to evaluate the significance of the length of BNN-20 administration with a starting point at P14. To do so, we administered BNN-20 in two schemes: from P14 to P40 and from P14 to P60. As expected, we documented further progressive loss of TH+ neurons in the “wv” SNpc: 41% at P14 that increased to 50% by P40 and reached 68% by P60 (Fig. 1A, B). Daily administration of BNN-20 during P14–P40 not only completely abolished further degeneration, but also significantly increased TH+ cell numbers in the SNpc by 26%, in respect to the P14 starting point, indicating the possible induction of a neurogenic process. Nevertheless, the numbers of TH+ neurons in the “weaver” SNpc remained significantly depleted (by approximately 29%), compared to age-matched wild-type (WT) mice (Fig. 1A, B). Longer BNN-20 administration (P14–P60) was equally efficient at blocking further degeneration and led to a slight increase in the number of TH+ neurons (in the wv BNN-20 P14–P60 SNpc compared to wv P14), although this increase was not statistically significant. TH+ cell number in the wv BNN-20 P14–P60 SNpc remained significantly reduced by 32% compared to age-matched wild-type mice (Fig. 1A, B). Administration of BNN-20 in WT mice had no impact on TH+ cell numbers of the SNpc (Additional File 3).

### Low-level dopaminergic neurogenesis exists in the SNpc of wild-type and “weaver” mice and is significantly enhanced by BNN-20

**Fig. 2:**
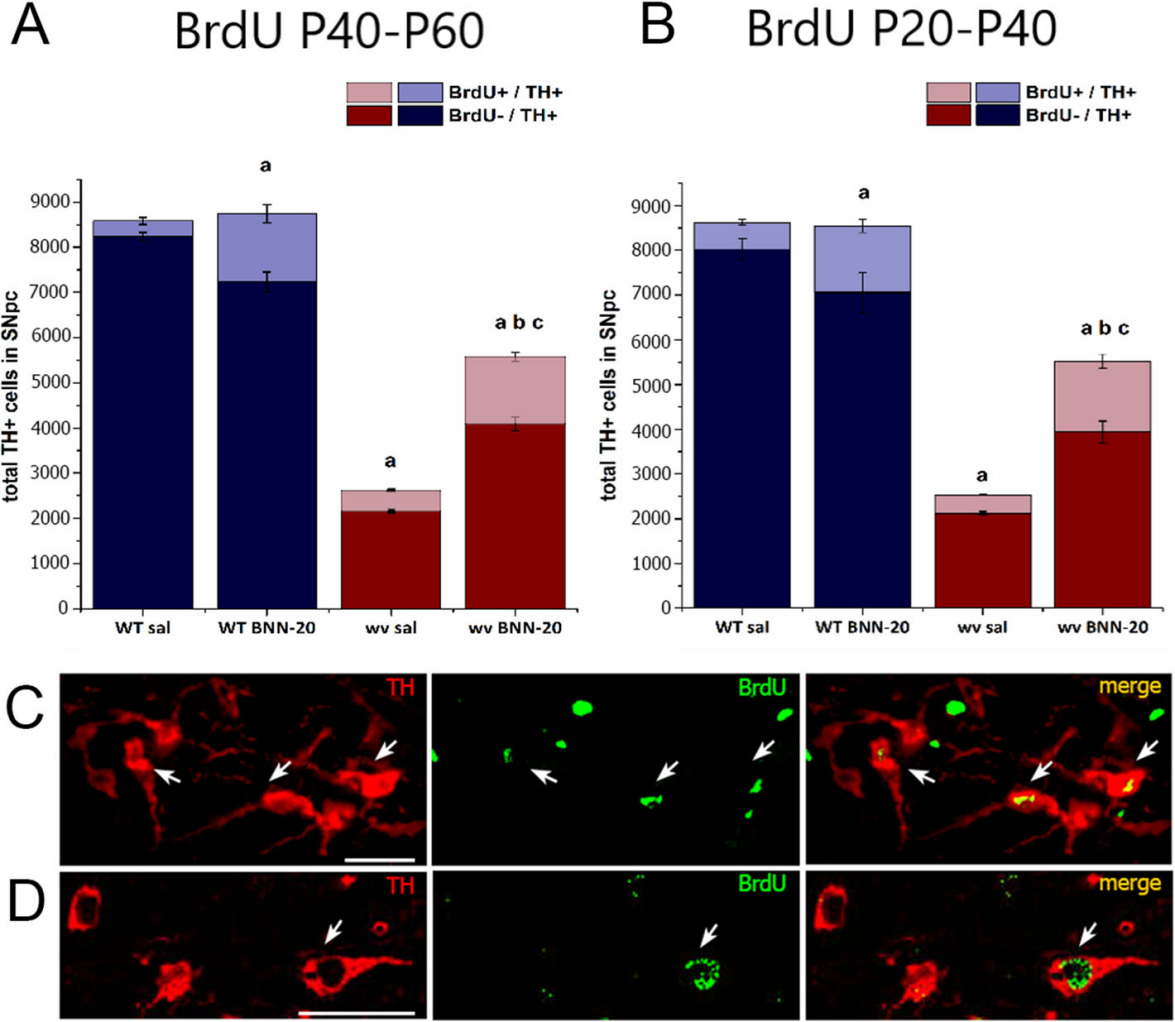
Newborn TH+/BrdU+ neurons in the postnatal WT and wv mouse SNpc; effects of BNN-20. **A,B** Stereologically calculated numbers of total TH+ cells in the SNpc of wild-type (WT) and “weaver” (wv) mice that received BNN-20 or saline (sal) from P14 to P60, with co-administration of BrdU from P40 to P60 (in **A**), or from P20 to P40 (in **B**). Co-expression of BrdU is also shown (TH+/BrdU+ cells, in light blue/pink and TH+/BrdU− in dark blue/red) [Graph **A**: *n* = 3 per group, TH+/BrdU+ to total TH+ percentage comparison: a: *p* < 0.01 compared to WT sal. b: *p* < 0.05 compared to wv sal. c: *p* < 0.05 compared to WT BNN-20. Graph **B**: *n* = 3 per group, a: *p* < 0.001 compared to WT sal. b: *p* < 0.001 compared to wv sal. c: *p* < 0.001 compared to WT BNN-20. Statistical analysis was performed by two-way ANOVA followed by Bonferroni post hoc test (*p* < 0.001, F = 41.993 for drug, *p* < 0.001, F = 42.679 for genotype). Error bars are SEMs]. **C,D** Representative images after immunofluorescence stainings against TH and BrdU in the SNpc of **C** a cluster of TH+/BrdU+ cells observed in the SNpc of wv BNN-20 mice (fluorescence microscopy) and **D** in the SNpc of WT BNN-20 mice (confocal microscopy). Double-positive (TH+/BrdU+) newborn dopaminergic neurons are indicated by white arrows [scale bar= 50 μm]

In order to investigate if the appearance of increased numbers of dopaminergic neurons in the adult weaver SNpc after the administration of BNN-20 was the result of neurogenesis, we decided to use BrdU labelling. Initially, we administered BrdU for 7 days, but we failed to identify double-positive BrdU/TH cells (data not shown). This indicated that a longer pulse was necessary, as expected for the differentiation/ maturation of newly born neurons and confirmed the absence of acute BrdU incorporation in dopaminergic neurons that could imply cell repair [33]. Based on previously published long-term BrdU administration schemes [34], we chose to apply two distinct BrdU schemes, both involving daily administration of BrdU for 20 consecutive days (see Additional File 1). Initially, we administered BNN-20 or saline (sal) from P14 to P60 in WT and wv mice with co-administration of BrdU between P40 and P60. Our data regarding the total number of TH+ cells in all four experimental groups revealed no indication of BrdU-induced toxicity (compare Figs. 1 and 2A, B). The presence of newborn dopaminergic neurons (TH+/BrdU+) at P60 was evaluated by immunofluorescence (Fig. 2C) and confocal (Fig. 2D) microscopy. BrdU+/TH+ double-positive dopaminergic neurons were detected in the SNpc of all experimental groups, indicating the presence of adult dopaminergic neurogenesis in the SNpc (Fig. 2A, B). Interestingly, the administration of BNN-20 led to a significant rise in the appearance of newborn TH+ cells in the WT SNpc, increasing their average number from 346 ± 77.49 to 1389.7 ± 208.16 per SNpc, and their average contribution to the total TH+ cell population from 4.03 ± 0.09% to 17.28 ± 2.28% (Fig. 2A,B). However, despite this increase, BNN-20 did not affect the total TH+ cell number in the WT SNpc (Fig. 2A,B; total TH+ cell numbers in the WT SNpc post BNN-20 administration are provided as Additional Material—(See Additional File 3)).

In the wv sal SNpc, the absolute number of double-positive cells was similar to the one observed in the WT sal SNpc (414.72 ± 32.07 per SNpc on average), but their contribution to the depleted pool of dopaminergic neurons was dramatically increased, making on average the 17.43 ± 1.28% of all TH+ cells (Fig. 2A). More importantly, BNN-20 administration significantly enhanced the presence of new dopaminergic neurons in the wv SNpc, increasing their average number to 1357.44 ± 100.68 and their contribution to 26.69 ± 2.02% of all TH+ cells (Fig. 2A).

In order to investigate whether newborn neurons survive longer, we applied a second BrdU protocol, with BNN-20/sal administered from P14 to P60 (as previously) and co-administration of BrdU again for 20 days, but this time between P20 and P40 (see Additional File 1). Mice were sacrificed at the same time-point (P60), allowing a 20-day BrdU withdrawal period. In agreement with our previous findings, newborn dopaminergic neurons were detected in the SNpc of all experimental groups with their absolute numbers and fraction per total TH+ cells being at similar levels to the 1st protocol (Fig. 2B).

### The newborn dopaminergic neurons follow the canonical SNpc differentiation pathway, expressing FoxA2

**Fig. 3:**
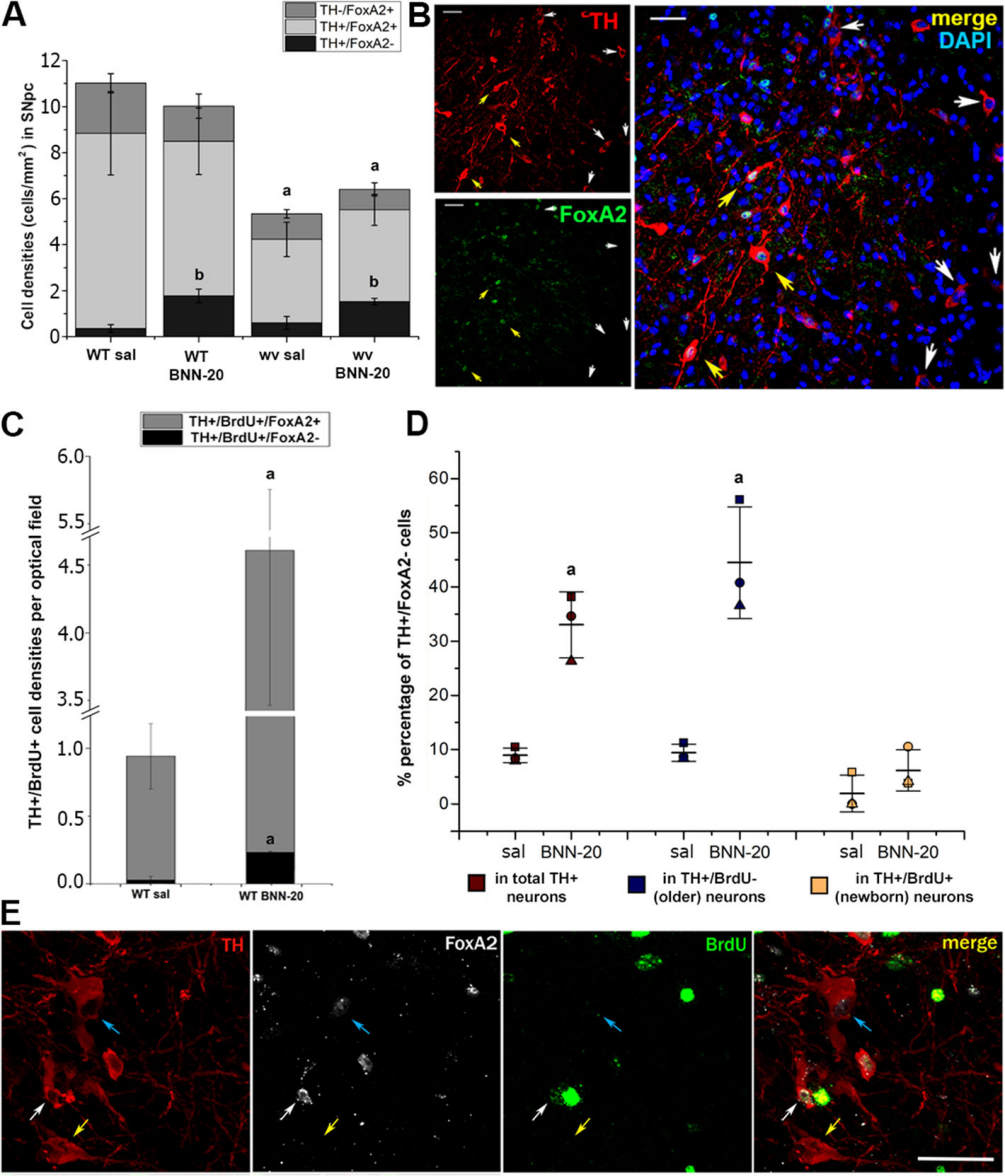
FoxA2 expression in the dopaminergic cell lineage of the SNpc. **A** Cell densities of the different subpopulations within the dopaminergic lineage (based on expression of TH and FoxA2) **A** in the SNpc of wild-type (WT) and “weaver” (wv) mice that received BNN-20 or saline (sal) from P14 to P60 and **Β** a characteristic example of immunostaining (with some of the TH+/ FoxA2− cells indicated by white arrows, and some of the TH+/FoxA2+ cells indicated by yellow arrows) [scale bar= 50 μm. a: decreased total TH+ cell densities (*p* < 0.05) compared to WT BNN-20 and WT sal groups (F = 7.94, *p* = 0.023 for genotype); b: increased TH+/FoxA2− densities (*p* < 0.05) compared to WT and wv sal groups (F = 25.971, *p* = 0.001 for drug), using two-way ANOVA followed by LSD post hoc analysis. *n* = 3 animals per group. Error bars are SEMs.]. **C** TH+/BrdU+ cell densities per optical field in the SNpc of the WT sal and WT BNN-20 groups, also showing the co-expression of FoxA2 [a: increased TH+/BrdU+/FoxA2+ and TH+/BrdU+/FoxA2− densities compared to WT sal; *p* < 0.05, using Student’s t test, *n* = 3 animals per group. Error bars are SEMs]. **D** Percentage of the newly described TH+/FoxA2− cells within the pools of total TH+ cells (in dark red), older (TH+/BrdU−, in blue), and newborn (TH+/BrdU+, in yellow) dopaminergic neurons in the WT SNpc post-administration of BNN-20 or saline (sal) (P14–P60) [a: increased % TH+/FoxA2− percentage compared to WT sal; *p* < 0.05, using Student’s t test, *n* = 3 animals per group. Error bars are SDs]. **E** A characteristic example of immunostaining indicating the 3 dopaminergic subpopulations of the SNpc: TH+/BrdU+/FoxA2+ (white arrow), TH+/BrdU−/FoxA2+ (cyan arrow), and TH+/BrdU−/FoxA2− (yellow arrow) [scale bar= 50 μm]

Dopaminergic neurons in the SNpc express TH, as well as specific transcription factors such as Nurr1, FoxA2, and Pitx3 that are necessary during specification, maturation, and survival. Notably, FoxA2 is considered to be a crucial transcription factor for the acquisition, function, and preservation of the mesencephalic dopaminergic phenotype [35, 36]. We assessed cell-type profiles in the SNpc of all experimental groups using high-magnification immunofluorescence analysis for TH and FoxA2 (Fig. 3A, B). In WT mice, we identified the typical dopaminergic neurons (TH+/FoxA2+) complemented by a pool of FoxA2+ only immunopositive cells and a small, previously undescribed population of TH+/FoxA2- neurons accounting for 5.12 ± 3.36% and 12.62 ± 3.54% of the total TH+ cells in the SNpc of WT sal and wv sal SNpc, respectively (Fig. 3A,B). In wv mice, both the density of total TH+ and of TH+/ FoxA2+ cells were significantly reduced (Fig. 3A), while the density and the percentage of FoxA2 + only cells (Fig. 3A and data not shown**)** remained at normal levels. Interestingly, the administration of BNN-20 significantly increased the density of TH+/FoxA2− cells in the SNpc of both the WT (4.2-fold) and wv (2.3-fold) mice, to 21.48 ± 3.29% and 29.08 ± 1.35%, respectively (Fig. 3A,B).

Notably, almost all newborn dopaminergic neurons (TH+/BrdU+) were also FoxA2+, with an average of 98.04 ± 1.96% and 93.82 ± 2.18% of TH+/BrdU+ neurons in the WT sal and WT BNN SNpc, respectively, being FoxA2+ (Fig. 3C–E).

This result implies that newborn dopaminergic neurons (irrespective of whether they have been induced by BNN-20) follow the canonical pathway for the SNpc differentiation and express FoxA2.

In contrast, the percentage of FoxA2− cells in both the total TH+ and the older TH+/BrdU− cell subpopulations is higher, with BNN-20 administration significantly increasing their percentage in the older (TH+/BrdU−) cells (from 9.43 ± 1.56% to 44.48 ± 10.28% on average) (Fig. 3D).

As the population of TH+/FoxA2− cells has not been reported before, we compared their morphology with that of the typical dopaminergic neurons of the SNpc (TH+/FoxA2+) and of the TH+/FoxA2− cells of the olfactory bulbs, a cell population constantly renewed by adult neural stem cells (Additional File 4). All three groups of cells were of a broadly similar elongated, pyramidal morphology, with one long dimension parallel to the coronal level and two shorter perpendicular dimensions, the shortest along the sagittal level. The ratio of the three dimensions was on average 3.9:1.7:1 for typical dopaminergic neurons in the SNpc, 3.2:1.9:1 for the TH+/FoxA2− cells of the Nigra and 3.0:2.1:1 for the TH+ interneurons of the OBs. One-way ANOVA for each dimension revealed that each cell group differed significantly from the other two at the longest and the shortest dimensions, with OB cells being always the smallest and TH+/FoxA2− significantly smaller than TH+/FoxA2+ cells (Additional File 4A-F).

### BNN-20 has no effect in stem and progenitor cells of the major neurogenic niches of the adult mouse brain

**Fig. 4:**
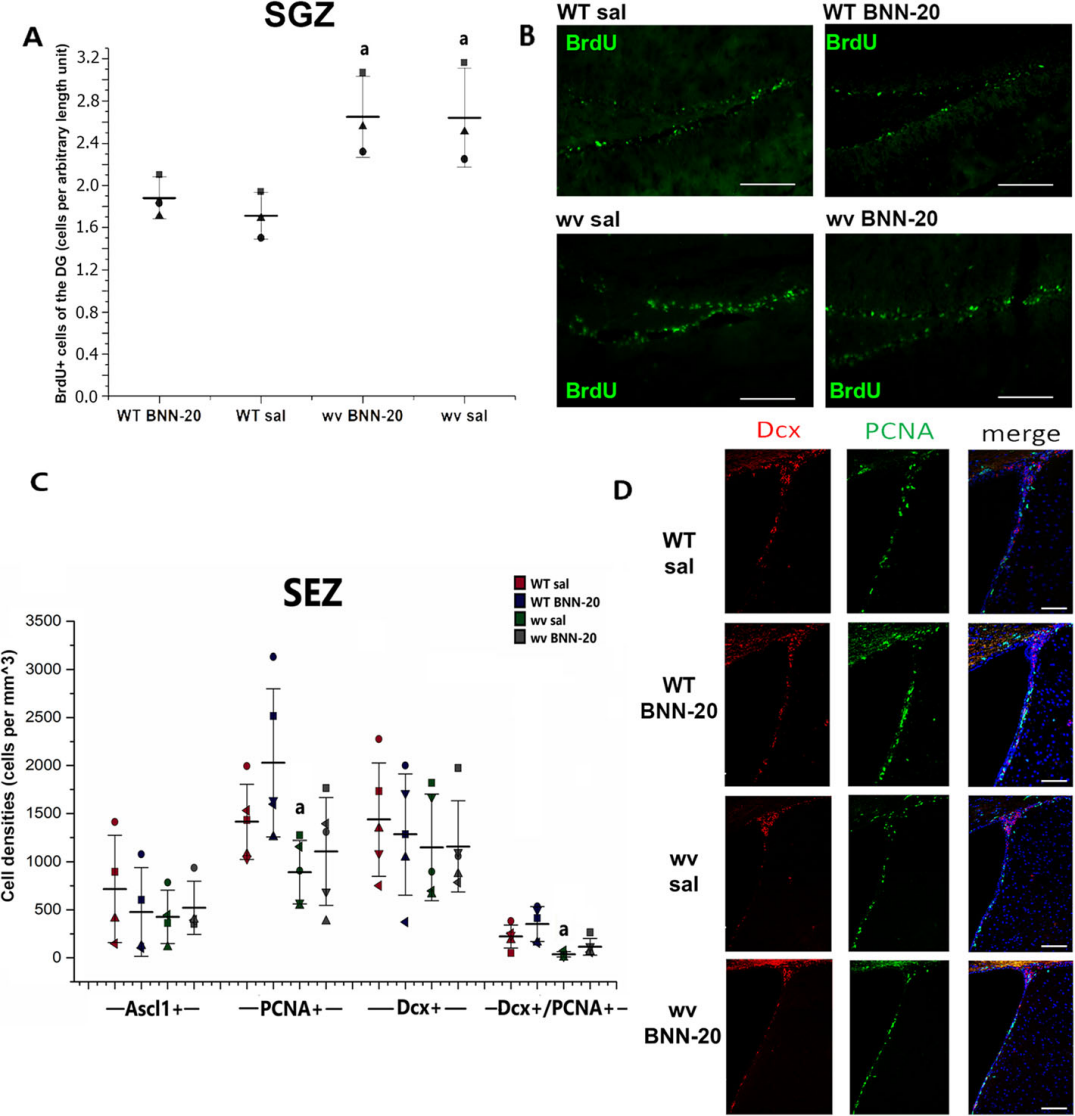
Lack of effect of BNN-20 administration on the main neurogenic zones of the postnatal brain. **A** Dot plot of numbers of BrdU+ cells per length and **B** representative immunofluorescence images of BrdU+ cells in the subgranular zone (SGZ) of the dentate gyrus (DG) of the hippocampus [*n* = 3 per group. Scale bar 200 μm. Error bars are SDs. a: *p* < 0.05 compared to the respective WT group, using two-way ANOVA (*p* = 0.002, F = 18.881) followed by LSD post hoc test]. **C** Histogram of the cell density of transient neural progenitors (Ascl1), proliferating cells (PCNA+), neuroblasts (Dcx+), and Dcx+/PCNA+ cells in the SEZ [*n* = 2 mice per group. a: *p* < 0.05 compared to WT sal. Analysis was performed by two-way ANOVA (PCNA+ comparison: *p* = 0.009, F = 8.902 for genotype, PCNA+/Dcx + comparison: *p* = 0.001, F = 18.030 for genotype) followed by LSD post hoc test. Error bars are SDs.]. **D** Representative immunofluorescence images of PCNA+ and Dcx+ cells within the SEZ of WT or wv mice post-administration of BNN-20 or saline (sal) during P14–P60 [scale bars = 200 μm]

Our findings suggested the existence of neurogenic activity within the SNpc, which is not a typical neural stem and progenitor cell (NSPC) niche of the postnatal mouse brain. We, therefore, complemented these observations, by assessing the possible effects of BNN-20 on endogenous pools of NSPCs, residing in the major cytogenic niches located in the subependymal and subgranular zones [37]. We counted the number of BrdU+ cells in the subgranular zone (SGZ) of the dentate gyrus, in the hippocampus of WT and wv animals that received BNN-20/sal from P14 to P60 with co-administration of BrdU during P20-P40. BrdU+ cells were significantly increased by approximately 50% in the wv SGZ, compared to age-matched WT mice (Fig. 4A, B); however, the administration of BNN-20 had no effect on the numbers of BrdU+ cells in both genotypes.

In the SEZ, neurogenesis as judged by the density of transit amplifying progenitors (Ascl1+ cells) and of neuroblasts (doublecortin+ cells) remained at normal levels in the wv mice (compared to WT), and after the administration of BNN-20 (Fig. 4C, D). Proliferation (PCNA+ cells) was significantly reduced in the wv SEZ, especially in the pool of neuroblasts (PCNA+/Dcx + cells), with BNN-20 administration having no effect on proliferation of both WT and wv NSPCs (Fig. 4C).

### The subependymal zone niche contributes to dopaminergic neurogenesis of the SNpc

**Fig. 5:**
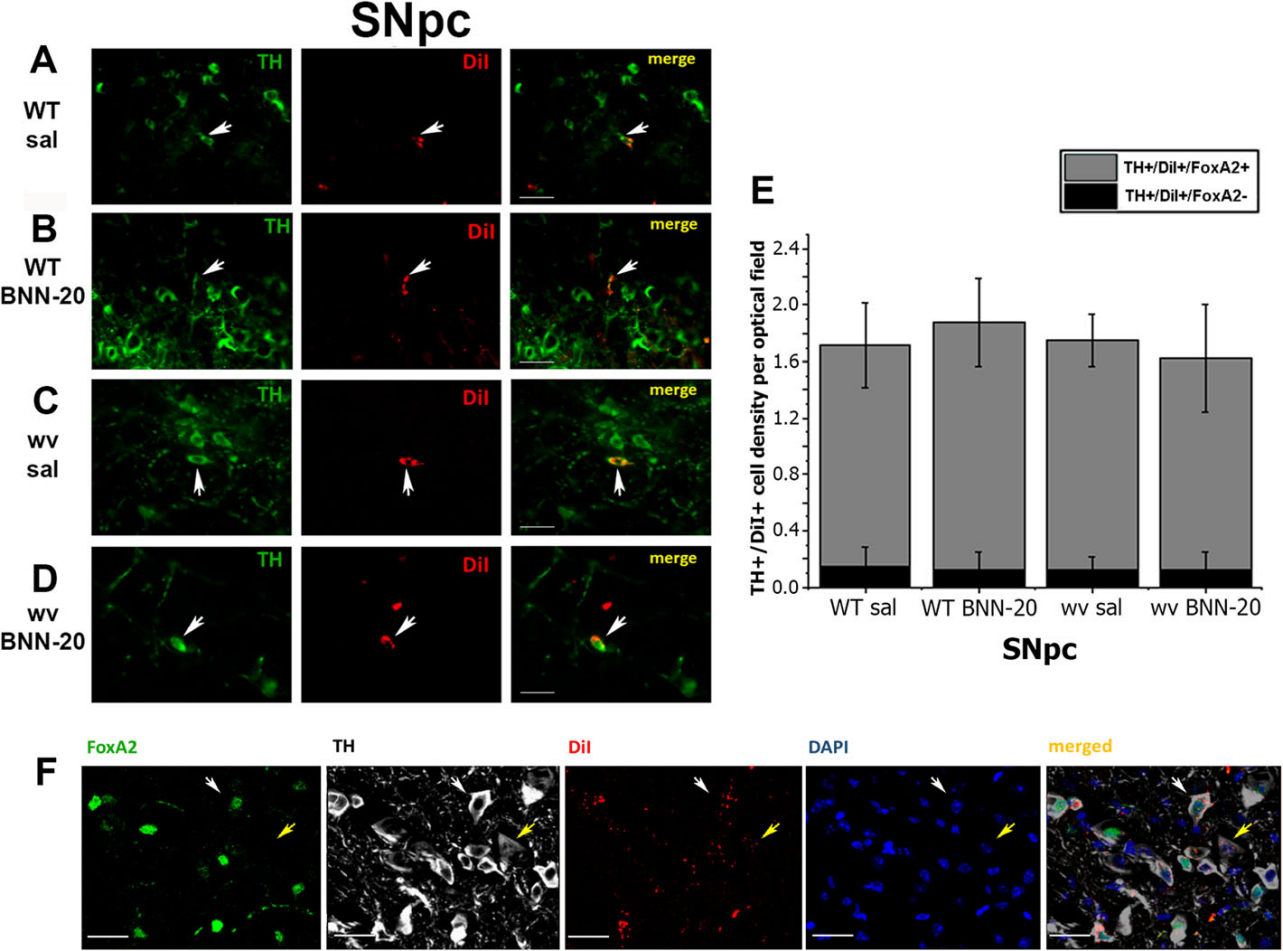
Contribution of the SEZ in dopaminergic neurogenesis in the SNpc. **A–D** Characteristic images of DiI+/TH+ newborn dopaminergic neurons in the SNpc (indicated by white arrows), derived from the SEZ neurogenic niche of the adult brain [scale bar = 50 μm]. **E** Profile of DiI+ dopaminergic (TH+) neurons of the SNpc in wild-type (WT) and “weaver” (wv) mice, after BNN-20 [or saline (sal)] administration, based on co-expression of FoxA2 [*n* = 2 mice per group. Error bars are SEMs. Statistical analysis was performed using two-way ANOVA followed by Bonferroni post hoc test]. **F** Representative triple immunofluorescence image depicting a cluster of DiI+/TH+ neurons in a WT BNN-20 SNpc. The white arrow indicates a TH+/DiI+/FoxA2+ cell and the yellow arrow a TH+/DiI+/FoxA2− dopaminergic neuron [scale bar = 50 μm]

Based on previously published data [25, 27], we investigated the origin of newborn dopaminergic neurons by labelling and tracing SEZ-derived cells, using unilateral intracerebroventricular injections of DiI on P45. DiI was evenly distributed on the ventricular walls [Images of DiI labelling throughout the ventricular system are provided as Additional Material (See Additional File 5)], including the domains where NSPC populations reside. 15 days post injection (P60), we were able to detect double-positive DiI+/TH+ neurons in the SNpc of all experimental groups (Fig. 5A–D). This finding strongly indicates that the SEZ contributes newborn dopaminergic neurons to the SNpc. The majority of DiI+/TH+ cells in the SNpc co-expressed FoxA2, suggesting that the SEZ-derived newborn neurons are following the typical developmental pathway of nigral dopaminergic neurons (Fig. 5E, F). Moreover, similarly to what we observed for the BrdU+/TH+ pool of newborn dopaminergic cells in the SNpc (Fig. 3B, C), the fraction of TH+/FoxA2− neurons within the pool of SEZ-derived DiI+/TH+ cells was very low, compared to the average percentage of TH+/FoxA2− cells within the total pool of TH+ cells in the SNpc, and was not affected by BNN-20 administration, with the average percentage of DiI+/TH+ neurons that were also FoxA2+ being in the range of 91.67–93.33% between groups (Fig. 5E, F).

### BNN-20 enhances neurogenesis in SN-derived primary NSPC cultures

**Fig. 6:**
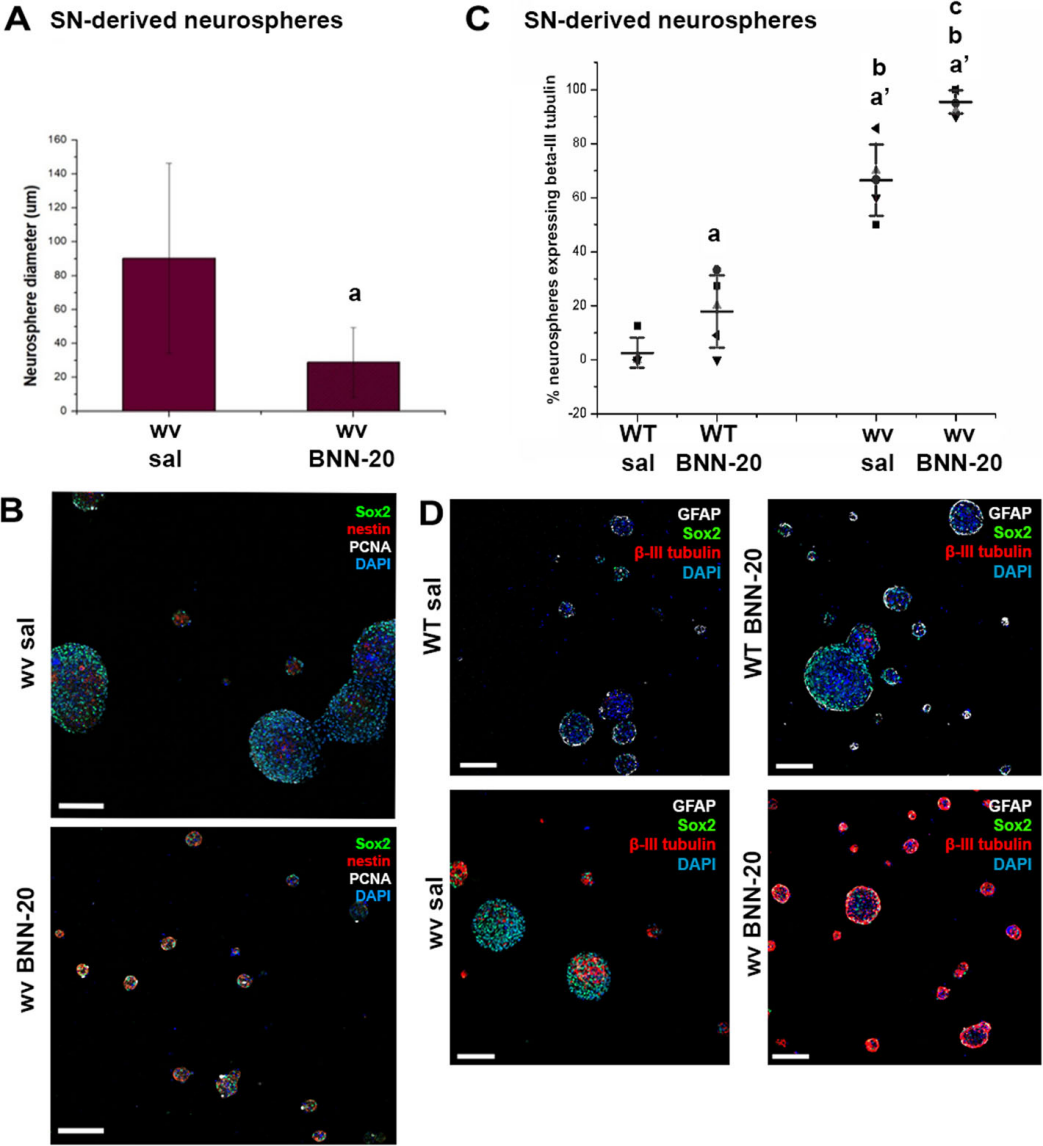
In vivo BNN-20 administration enhances differentiation of isolated NSCs into neurons, in primary cell cultures. **A** Histogram showing the average diameter of primary neurospheres generated from the SN of weaver mice having received saline or BNN-20 (P14–P60) [a: *p* < 0.05 compared to wv sal. Analysis was performed using Student’s t test, *n* = 5 optical fields per group. Error bars are SEMs.]. **B** Representative images after immunofluorescence staining for the NSPC markers Sox-2 (green) and nestin (red), as well as for the proliferation marker PCNA (white) [scale bars = 100 μm]. **C** Dot plot showing the percentage of SN-derived primary neurospheres containing at least one cell expressing the neuronal marker β-ΙΙΙ tubulin. Neurospheres were generated from SN of wild-type (WT) and “weaver” (wv) mice that had received BNN-20 or saline (sal) (P14–P60) [Error bars are SDs. a: *p* < 0.05 & a’: *p* < 0.01 compared to WT sal. b: *p* < 0.01 compared to WT BNN-20. c: *p* < 0.01 compared to wv sal. Analysis performed using two-way ANOVA followed by LSD post hoc test (*p* < 0.001, F = 24.207 for drug, *p* < 0.001, F = 245.576 for genotype, *n* = 5 optical fields per group)]. **D** Representative images after immunofluorescence staining for the NSPC marker Sox-2 (green) and the differentiation markers GFAP (white) and βIII tubulin (red) [scale bars= 100 μm]

Postnatal brain NSPCs can be isolated and cultured in the form of 3D cell aggregates called neurospheres. In order to investigate the effects of BNN-20 on NSPCs, we used an ex vivo approach by isolating NSPCs from the SN of WT and wv (Ρ90) mice that had received BNN-20/sal between P14 and P60. Primary neurospheres were generated and we performed immunofluorescence analysis for proliferation, progenitor identity, and differentiation markers (Fig. 6).

Neurospheres derived from the SEZ or the SN of WT animals were of similar size irrespective of prior BNN-20 administration (data not shown). However, neurospheres grown from the SN of wv mice that had received BNN-20 were significantly smaller, compared to those grown from wv mice having received saline (Fig. 6A, B). Primary neurospheres were immunostained for β-ΙΙΙ tubulin (to mark immature neurons), GFAP (to mark astrocytes), and Sox2 (to mark NSPCs). As expected, spontaneously differentiated βIII tubulin+ cells were almost undetectable in WT SN-derived neurospheres grown in proliferation medium (Fig. 6C, D). Interestingly, the percentage of neurospheres containing at least one such β-ΙΙΙ tubulin+ cell was significantly increased, almost 28-fold (to 66.48 ± 5,90%), in the wv SN cultures (Fig. 6C, D) compared to WT, while the administration of BNN-20 led to a significant increase in the fraction of neurospheres containing β-ΙΙΙ tubulin+ cells in both WT and wv mice. Impressively, almost all primary SN neurospheres derived from wv mice that had received BNN-20 included β-ΙΙΙ tubulin+ cells (95.46% ± 2.01%) (Fig. 6C, D).

**Fig. 7:**
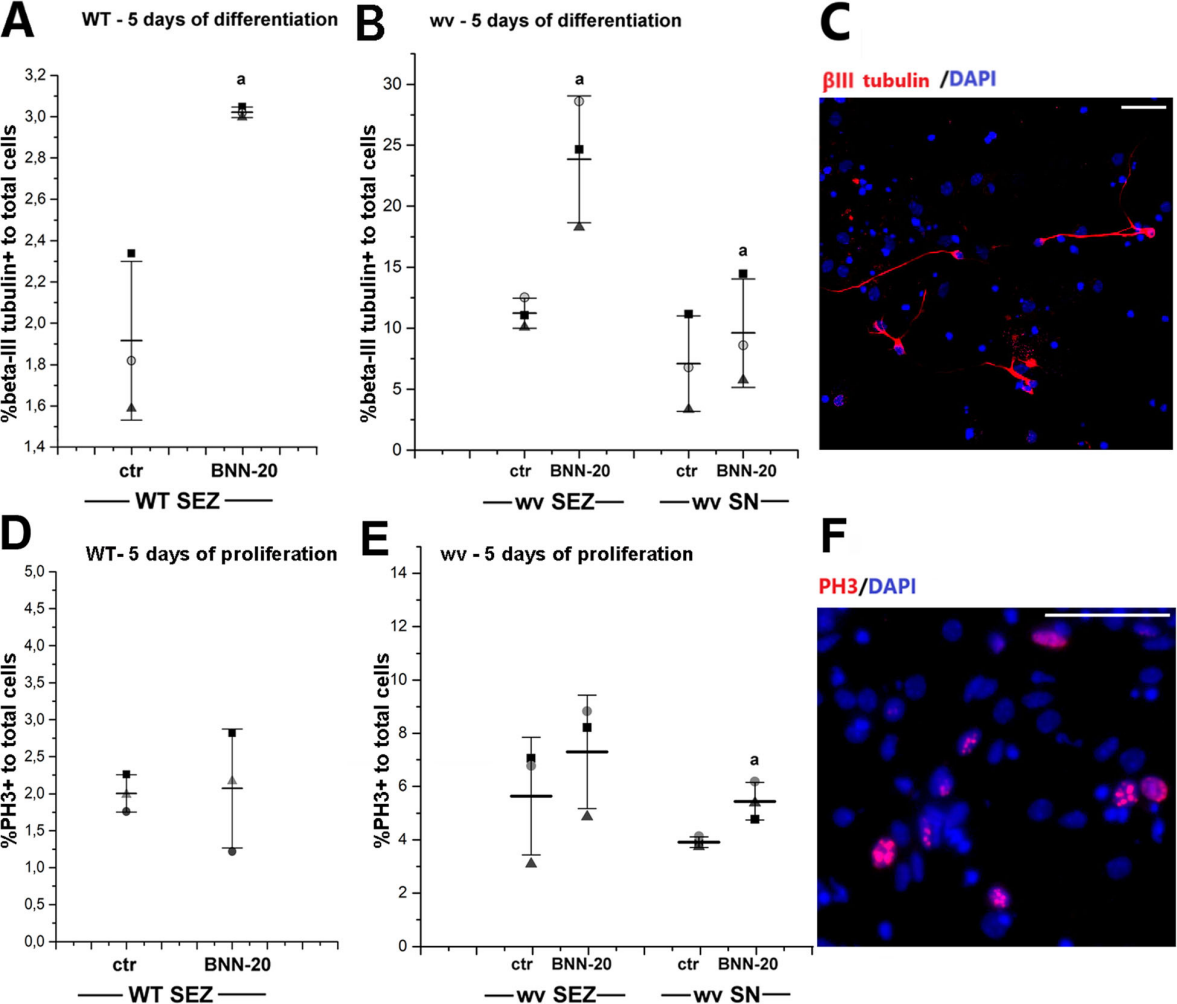
BNN-20 promotes neuronal differentiation of NSCs in vitro*.* **A,B** Dot plots showing the percentages of β-ΙΙΙ tubulin+ neurons in cultures of **A** wild-type (WT) SEZ-derived NSCs and **B** weaver (wv) SEZ- and SN-derived NSCs maintained in differentiation conditions for 5 days with or without (ctr) BNN-20 addition in the cell medium. **C** Representative immunofluorescence image of a cell culture stained for βIII tubulin [scale bar = 50 μm]. **D,E** Percentages of phosphohistone 3+ (PH3+) cells in cultures of **D** wild-type (WT) SEZ-derived and **E** weaver (wv) SEZ- and SN-derived NSCs, maintained for 5 days in proliferation conditions with or without (ctr) BNN-20 addition in the cell medium [Error bars are SDs. a: *p* < 0.05 using paired Student t-test analysis because the same cell samples were split in ctr or + BNN-20 conditions. *n* = 3 independent experiments]. **F** Representative immunofluorescence image of a cell culture stained for PH3 [scale bar= 50 μm]

In order to investigate whether the pro-neurogenic effect of BNN-20, documented in vivo and ex vivo, was direct on NSPCs or indirect (i.e., mediated by BNN-20 altering the microenvironment of the SN [13], we decided to culture SEZ and SN-derived neurospheres (from untreated animals) with or without the presence of BNN-20 in the medium. In order to eliminate any “memory” of the in vivo microenvironment, in these cell assays we used neurospheres that had been passaged at least 4 times (> P4 < P8). The few neurospheres grown from WT SN could not be passaged more than once; thus, they could not be included in this line of experiments. In SEZ-derived WT neurospheres, the addition of BNN-20 in the medium during 3, 5, or 7 days of differentiation (dd) resulted in a significant increase (consistently higher than 50%) in the appearance of Dcx + immature neurons (Additional File 6A, C), as well as in a significant increase in GFAP+ astrocytes (Additional File 6B, D). Moreover, the addition of BNN-20 in the medium during 5dd also resulted in a significant increase (of 61.7 ± 16.98%) in the appearance of more mature β-ΙΙΙ tubulin+ neurons (Fig. 7A). Exposure to BNN-20 for 5 days in proliferation conditions had no effect on WT SEZ-derived NSPCs, as judged by the percentages of cells immunopositive for proliferation markers Ki67 (data not shown) and phosphohistone 3 (PH3) (Fig. 7D).

In contrast to WT mice, wv SN-derived neurospheres could be efficiently passaged, and thus used for investigating the in vitro effects of BNN-20 along with SEZ-derived cultures. Similarly to what we saw in the WT NSPC cell cultures, BNN-20 addition in the cell culture medium for 5 days of differentiation also led to a significant increase in the appearance of β-ΙΙΙ-tubulin+ neurons, both in wv SEZ (by 110.8 ± 14.7%) and SN-derived (by 42.63 ± 14.69%) neurospheres (Fig. 7B). Addition of BNN-20 for 5 days in the proliferation medium did not change the presence of PH3+ cells in wv SEZ-derived neurospheres, but significantly increased the percentage of PH3+ cells in SN-derived neurospheres (Fig. 7E).

These results indicate that BNN-20’s neurogenic effect is mainly attributed to its ability to promote the differentiation of NSPCs towards neurons and, to a much lesser extent, to any effects in NSPC proliferation.

### BNN-20 increases the presence of newborn NSPCs in the wild-type SNpc without affecting astrogliogenesis

**Fig. 8:**
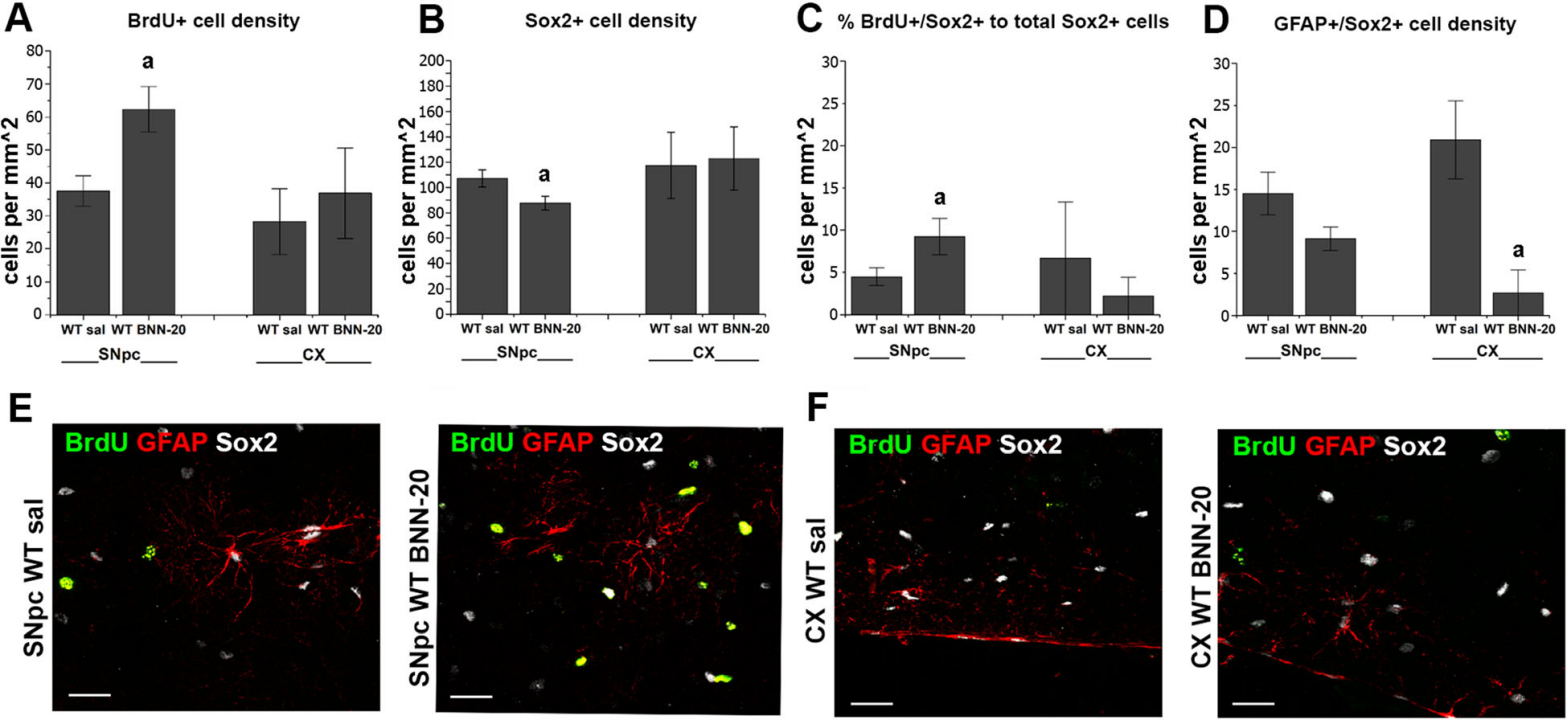
No effect of BNN-20 on BrdU+ cells in the cortex and on astrogliogenesis. **A** Cell densities of total BrdU+ cells in the SNpc and cortex (CX) of wild-type (WT) animals after administration of BNN-20 or saline (sal) (P14–P60) [a < 0.01 compared to WT sal]. **B** Cell densities of total Sox2 expressing cells in the SNpc and CX WT animals after administration of BNN-20 or sal (P14–P60) [a < 0.01 compared to WT sal]. **C** Percentage of newborn (BrdU+/Sox2+) to total Sox2 expressing cells in the WT SNpc and the WT CX after administration of BNN-20 or saline (sal) (P14–P60) [a < 0.05 compared to WT sal]. **D** Cell densities of total GFAP+/Sox2+ expressing cells in the WT SNpc and the WT CX after administration of BNN-20 or saline (sal) (P14–P60) [a < 0.05 compared to WT sal]. Statistical analyses for the data presented in histograms **A–D** were performed using Student’s t test. *n* = 3 animals per group. Error bars are SEMs. **E,F** Representative immunofluorescence images of triple stainings against BrdU (green), GFAP (red), and Sox2 (white) in **E** the SNpc and **F** the CX of WT animals that received saline (sal) or BNN-20 (P14–P60) [scale bars= 50 μm]

Our data suggested that BNN-20 was able to induce a neurogenic response in normal WT mice and that this response was taking place in the SNpc, outside the canonical neural stem cell niches. In order to investigate further these unexpected results, we performed additional immunohistochemical analyses in WT mice that had received BNN-20 (P14–P60) in combination with BrdU pulses (P20–P40). We performed co-stainings for BrdU and for Ascl1 to identify transit amplifying progenitors (the direct daughter cells of neural stem cells in the SEZ) [38], or for Sox2 (a key transcription factor expressed in NSPCs and activated astrocytes) and GFAP. We focused our analysis in the SNpc and the cerebral cortex (area V2MM), a structure known to harbor latent NSPCs [39, 40]. In saline-treated WT mice, BrdU+ cells were detected in the SNpc and the cortex (Fig. 8A). We could not detect any Ascl1 immunopositive cells outside the SEZ and a population of Sox2+ cells of equal density was present in the two areas (Fig. 8B). After administration of BNN-20, we confirmed a significant increase in the density of BrdU+ cells in the SNpc, which was not detected in the cortex (Fig. 8A). BNN-20 did not lead to the appearance of Ascl1+ cells in either area, but had a dual effect on Sox2+ cells, only in the SNpc. It led to a significant decrease in their total density (Fig. 8B) but increased the percentage of newborn Sox2+ cells (co-expressing BrdU) in the total Sox2+ cell pool by 104.66% (Fig. 8C), hence, confirming a pro-differentiation effect combined with a sustained progenitor pool in the affected SNpc, respectively. Astrogliogenesis was very low in the WT SNpc and cortex, as we failed to detect double BrdU+/GFAP+ and the density of Sox2+/GFAP+ cells that would mark activated or immature astrocytes was decreased after BNN-20 administration, this being significant in the cortex (Fig. 8D). Overall, this analysis revealed a dual specificity in the effects of BNN-20: the microneurotrophin led to an increase in BrdU+ cells in the SNpc and not in the cortex, without enhancing astrogliogenesis.

## Discussion

Recruiting endogenous NSPCs of the adult brain, rather than resorting to transplantations or in vivo cell reprogramming [4–6] for the treatment of neurodegenerative diseases, such as Parkinson’s disease, in which selective replacement of neural cells can be effective for [41, 42], would be highly desirable. However, the existence of endogenous dopaminergic neurogenesis in the SNpc that could be targeted in cases of PD remained poorly investigated and controversial, with few research groups reporting the detection of adult-born dopaminergic neurons in healthy mice and rats [24, 27, 43], and few others failing to reproduce these results [22–24, 26, 44, 45]. Similarly contradictory results have been reported for the induction of dopaminergic neurogenesis in the SNpc in chemotoxic parkinsonian rodent models, such as 6-OHDA and MPTP [26, 44, 45].

Here, we provide strong evidence of dopaminergic neurogenesis in the postnatal wild-type mouse brain and in the “weaver” mouse, a genetic model of gradual nigrostriatal dopaminergic degeneration. We employed laborious BrdU-based investigation of cytogenesis in the SNpc, combined with cell-type profiling for NSPCs and for cells of the dopaminergic lineage, as well as cell culture assays. Our key findings are that a constant turnover of dopaminergic neurons exists in the normal postnatal SNpc and that this system can be pharmacologically manipulated with clinically relevant specificity and efficiency, in order to partially avert the progressive degeneration of the SNpc in the “weaver” mouse.

### Characterization of SNpc neurogenesis

Using long BrdU administration and exhausting confocal microscopy analysis, we showed that newborn dopaminergic neurons appear in the normal postnatal SNpc, following a canonical mesencephalic developmental pathway (expressing the key transcription factor FoxA2). This is most interesting because by using DiI i.c.v injections, we showed that NSPCs of the SEZ niche contribute to this process. These NSPCs are known to generate TH+ interneurons in the olfactory bulbs (OB) [46] that we showed to lack expression of FoxA2 and to have distinct morphology to the typical nigral TH+/FoxA2+ cells. Hence, SEZ cells are able to adjust remarkably to local requirements, acquiring a mesencephalic fate in the SNpc or an oligodendroglial fate when recruited in sites of demyelination in the corpus callosum [47]. When the system was stimulated in the healthy brain, via the administration of BNN-20 in WT mice, neurogenesis in the SNpc was significantly increased, albeit without an expansion of the TH+ cell pool, possibly indicating a turnover/ rejuvenation mechanism, similar to what has been described in the OB and the hippocampus [46, 48–50].

The absolute numbers of newborn dopaminergic neurons (BrdU+/TH+) were found to be similar in the WT and the wv SNpc and, as a result, their representation in the degenerated wv SNpc was significantly increased. This suggests that the system of neurogenesis in the SNpc is insensitive to degeneration and at the same time indicates that even postnatally born dopaminergic neurons are initially resistant to degeneration, which appears later, in a time/maturation-dependent pattern. This is in contrast to previous findings in the 6-OHDA and MPTP parkinsonian models [24, 25, 27], in which an induction of dopaminergic neurogenesis was observed in the SNpc even in vehicle-injected mice [27]. These discrepancies could be attributed to differences in the models used (acute mechanical injury versus gradual degeneration) and the balance between addition and removal of cells, and indicate that the SNpc neurogenic system responds differently to different injuries.

An obvious concern is whether the generated TH+ cells are of functional significance. We have recently shown [15] that BNN-20 administration in the wv mouse, using the same administration scheme (P14–P60, 100 mg/kg b.w. daily, i.p.) that we show here to lead to the significant induction of dopaminergic neurogenesis in the SNpc, results in a 3-fold increase of dopamine levels in the striatum, as well as in significantly improved locomotor activity. Our results are further supported by recently published experimental work that showed very clearly that new nigral neurons, either grafted or generated via reprogramming [4, 5], can reconstruct the dopaminergic nigrostriatal connection.

A surprising finding was the detection of a, so far, unreported TH+ cell fraction, lacking expression of FoxA2. We showed that these cells are specifically under-represented in the pool of newly generated TH+ cells, identified as BrdU+ or DiI+, suggesting that this population mainly includes older dopaminergic neurons. That observation, combined with the marked increase of this cell type when generation of new cells in the wild-type SN is boosted by BNN-20, and the fact that their morphology differs from that of typical nigral dopaminergic neurons, led us to hypothesize that they could represent dopaminergic neurons that are about to be eliminated [having lost the expression of FoxA2 which is critical for the maintenance of the midbrain dopaminergic character [36]], in order to be replaced by newborn dopaminergic cells. Of course, further analysis is needed, in order to confirm this hypothesis. However, previously published experimental work has reported the existence of dying (TUNEL+) dopaminergic neurons in the wild-type SNpc, accompanied with no change in the total dopaminergic cell number [25].

Are all newly generated cells in the SNpc derived from the SEZ, as suggested by the DiI experiment? This question becomes highly relevant ahead of any prospect of transferring our results in the clinical practice, because the existence of adult SEZ neurogenesis in the human brain remains controversial. Several recent reports have indicated a rapid decline in the generation of SEZ-derived neuroblasts in the human brain after the 18th month of age [51, 52] as well as in the context of neurodegenerative disorders, including PD. [53–55] However, earlier work has shown that few of the surviving neuroblasts retain active proliferation [56] and can be recruited at remote areas of lesion, for example in cases of vascular dementia [57]. The capacity of the wv SN to generate self-renewing neurospheres, in contrast to the WT tissue, suggests that in the degenerative microenvironment, which is characterized by pro-neurogenic microglial activation [15], a pool of latent progenitors could become activated with a prominent neurogenic output, as has been previously reported for the injured cortex and midbrain [45, 58]. Notably, the presence of immature neurons (including TH+) has been reported in the SN of PD patients [59] and neural progenitor cells (NPCs) able of  in vitro proliferation and neuronal differentiation have been isolated from the SN of PD patients, postmortem [60]. Although we failed to detect Ascl1+ (a marker of non-migrating transit amplifying progenitors) cells in the SNpc, because Ascl1 is correlated with the generation of interneurons [61], its absence might be an additional evidence of the operation of midbrain-specific neurogenesis. On the other hand, we detected an increased percentage of newborn (BrdU+/Sox2) Sox2+ cells in the Sox2+ cell pool, after BNN-20 administration. Because expression of Sox2 is normally switched off when neuroblasts start to migrate out of the SEZ [32], its increased expression is compatible with the presence of activated local NSPCs.

### The beneficial effects of BNN-20

Our results identify the microneurotrophin BNN-20 as a potent modulator of neurogenesis in the SNpc. BNN-20 administration led to an impressive, 3-fold to 4-fold increase in the number of newborn dopaminergic neurons, in the SNpc of both “weaver” and WT mice. This neurogenic effect led to the partial restoration of the dopaminergic cell number in the degenerated wv SNpc, while it did not affect the total dopaminergic cell number in wild-types. Two of the most clinically relevant findings on BNN-20 are that its in vivo neurogenic effects are highly specific in terms of (i) region and (ii) lineage. It led to increased neurogenesis in the SNpc, without any effects in the SGZ and the SEZ niches, or in the cortex [that has previously been shown to harbor dormant NSPCs [58]], and it did not enhance astrogliogenesis.

BNN-20 exerts its beneficial effects acting as a mimetic of BDNF, and more specifically, by activating its selective receptor TrkB and the TrkB-PI2K-Akt-NFκΒ and TrkB-ERK1/2-NFκΒ downstream pathways [13, 15]. Co-administration of BNN-20 and ANA-12, a selective TrkB inhibitor, or the NFκB inhibitor Bay-11-7085, in “weaver” mice, not only abolished the BNN-20-dependent increase of BDNF in the SN, but also partially reversed the preservation of the dopaminergic neurons of the SNpc [13, 15]. BDNF has been shown to promote the proliferation, differentiation, migration, and survival of adult NSCs of the SEZ and the SGZ [62–66], even though there is still some controversy in the cell-type expression of the two isoforms of TrkB receptors (the full-length TrkB-FL and the truncated TrkB-T1) and the p75^NTR^ pan-neurotrophin receptor, as well as in the extent of each receptor’s contribution to BDNF’s effect on adult neurogenesis [38, 61, 64, 65, 67]. The full-length isoform of TrkB (TrkB-FL), which is able to activate the PI3K and MAPK downstream signaling cascades [48, 61, 65, 67, 68], is expressed by migrating neuroblasts [61, 65, 69] or the immature neurons of the OB [65, 67], and the p75^NTR^ receptor is expressed by intermediate progenitors (type C cells) of the SEZ and neuroblasts [65]. Hence, the effects of BDNF are mainly restricted on the migration, differentiation, and survival of newborn neurons, and not on the proliferation of the SEZ NSPCs [38, 65]. This is in accordance with our results that suggest BNN-20 acts mainly to enhance the differentiation of NSPC into neurons. Finally, these same signaling pathways (TrkB-PI3K-Akt and TrkB-ERK1/2) can “jump-start” ectopic neurogenesis in brain areas such as the striatum [64].

As mentioned earlier, using DiI tracing experiments, we have shown that at least part of the newborn dopaminergic neurons originates from the SEZ. However, we failed to detect any significant BNN-20 induced changes within the SEZ itself. These seemingly contradictory observations may be explained in two ways. Firstly, the neurogenic niche of the SEZ is an open system, where newborn neuroblasts quickly migrate out towards their target brain area [70]. Hence, a limited increase in the rate of differentiation of neural progenitors into neuroblasts that subsequently migrate towards the SNpc might not be easily detected in the SEZ. Secondly, the main effect of BNN-20 could be to enhance the differentiation/ survival of neuroblasts and of their progeny, specifically in the SNpc.

On the other hand, the SGZ is a closed system, with BDNF being a key regulating factor [65, 66]. Based on that, the lack of an effect after BNN-20’s in vivo administration on hippocampal neurogenesis is unexpected. However, there are some key differences between the SEZ and SGZ niches that might offer an explanation. The expression of neurotrophins (including BDNF) in the SEZ niche is low, when compared to the SGZ [65]. This means that a BDNF mimetic, such as BNN-20, is expected to evoke more easily an effect in the “neurotrophin poorer” SEZ niche, than in the “BDNF-saturated” SGZ. Furthermore, the expression patterns of the TrkB and p75^NTR^ receptors also significantly differ between the two niches, something that could also result in a differential activity of BNN-20 [65].

## Conclusions

Our results confirm and characterize the existence of dopaminergic neurogenesis both in the adult wild-type and the degenerated SNpc of the “weaver” mouse. Newborn dopaminergic neurons appear preferentially resistant to degeneration, as seen by their increased representation within the total TH+ cell pool of the wv SNpc, compared to WT. These newborn TH+ neurons originate, at least partially, from the neurogenic niche of the SEZ, although we offer evidence for the presence of local progenitors as well. Moreover, the newborn TH+/BrdU+ neurons of the SNpc follow the canonical differentiation pathway, expressing FoxA2 and are possibly functional. Long-term (P14–P40 or P14–P60) administration of the microneurotrophin BNN-20 leads to a significant enhancement (3-fold to 4-fold) of dopaminergic neurogenesis in the SNpc, in a tissue-specific manner, in both WT and wv mice. While this turnover supports the partial restoration of the dopaminergic cell number in the wv SNpc, it does not affect total TH+ cell numbers in the WT SNpc. Overall, BNN-20 is proved to be a multi-modal molecule, exhibiting beneficial effects through an antioxidant and neurotrophic activity [13], through the modulation of inflammation [15] and, as we demonstrate here, through the activation of endogenous NSPCs. The results presented here support BNN-20 as a promising drug candidate for future cell replacement therapies against PD, based on the manipulation of the SNpc’s endogenous regeneration capacity.

## Supplementary Information

**Additional file 1.** Graph depicting the administration schemes for BrdU and BNN-20, used for the *in vivo* labelling of the newborn TH+/BrdU+ neurons of the SNpc.

**Additional file 2.** The method used in morphological analysis of TH+ neurons. Three dimensions of dopaminergic neurons were analyzed: the longest dimension of the cell soma (in yellow), the perpendicular to the longest (in green) and the shortest (defined by the depth – z, as indicated at the lowest right corner) [Scale bars = 10 μm].

**Additional file 3.** BNN-20 administration has no effect on the total dopaminergic neuron number of the WT SNpc. Total dopaminergic (TH +) cell number in the SNpc of wild-type (WT) mice, untreated (WT P14, WT P40, WT P60), or treated with BNN-20 from P14 to P40 (P14-P40) or to P60 (P14-P60) [*n* = 4 per group. Error bars are SDs; statistical analysis was performed using two-way ANOVA, followed by the Bonferroni post-hoc test].

**Additional file 4.** Morphology of different dopaminergic cell populations. Morphological analysis of the 3 dopaminergic neuron populations shown in Fig. 3A, by comparison of: (A) the longest dimension, (B) the perpendicular to the longest dimension and (C): the shortest dimension (depth) of the cell soma. Details are shown in Additional File 2 [In A: a: *p* < 0.001 compared to SNpc TH+/FoxA2+ neurons, b: *p* < 0.05 compared to SNpc TH+/FoxA2- neurons. In C: a: *p* < 0.01 compared to SNpc TH+/FoxA2+ neurons, b: *p* < 0.01 compared to SNpc TH+/FoxA2- neurons. Error bars are SEMs. *n* = 10 dopaminergic neurons per group. Statistical analysis was performed using one–way ANOVA (*p* = 0.000, F = 24.551 in E; *p* = 0.000, F = 18.849 in G), followed by LSD post hoc analysis.]. (D-E) Characteristic immunofluorescence images of (D) a TH+/FoxA2+ neuron of the SNpc, (E) a TH+/FoxA2- neuron of the SNpc and (F) a TH+/FoxA2- neuron of the OB [Scale bar = 10 μm].

**Additional file 5.** DiI staining of the lining of the ventricular systems. DiI incorporation in the ependymal and subependymal layers of the lateral ventricles (LV), the third ventricle (3 V) and the Aqueduct (Aq) after one unilateral DiI injection in the left LV [Scale bars = 500 μm].

**Additional file 6.** BNN-20 promotes neuronal and astroglial differentiation of NSCs *in vitro* (additional info). (A,B) Dot plots showing the percentages of (A) Dcx + immature neurons and of (B) GFAP+ (astrocytes) cells in cultures of wild-type (WT) SEZ-derived NSCs maintained in differentiation conditions for (A) 3, 5, 7 or (B) 3 and 7 days with or without (ctr) BNN-20 addition in the cell medium [Error bars are SDs. a: *p* < 0.05 using paired Student t-test analysis because the same cell samples were split in ctr or + BNN-20 conditions]. (C-D) Representative immunofluorescence images of cell cultures stained for Dcx (in C) and GFAP (in D) [Scale bars = 50 μm. *n* = 3 independent experiments].

## Data Availability

The datasets used and/or analyzed during the current study are available from the corresponding author on reasonable request.

## Abbreviations

BBB: Blood-brain barrier
BDNF: Brain-derived neurotrophic factor
BrdU: 5′-Bromo-2′-deoxyuridine
b.w.: Body weight
DG: Dentate gyrus of the hippocampus
DMSO: Dimethyl sulfoxide
GFAP: Glial fibrillary acidic protein
MNT: Microneurotropin
NPCs: Neural progenitor cells
NSCs: Neural stem cells
NSPCs: Neural stem and progenitor cells
OB: Olfactory bulb
PD: Parkinson’s disease
PDL: Poly-d-lysine
PH3: Phosphohistone 3
SEZ: Subependymal zone of the lateral ventricles
SGZ: Subgranular zone of the dentate gyrus of the hippocampus
SN: The broader area of the substantia nigra (excluding all the periventricular areas)
SNpc: Substantia nigra pars compacta
TH: Tyrosine hydroxylase
WT: Wild-type mice
wv: Homozygous “weaver” mice
