# Supplementary figures and images for: Characterization of substantia nigra neurogenesis in homeostasis and dopaminergic degeneration: beneficial effects of the microneurotrophin BNN-20
Source: Stem Cell Res Ther. 2021 Jun 10;12:335. doi: 10.1186/s13287-021-02398-3 (PMC8193896; doi:10.1186/s13287-021-02398-3)

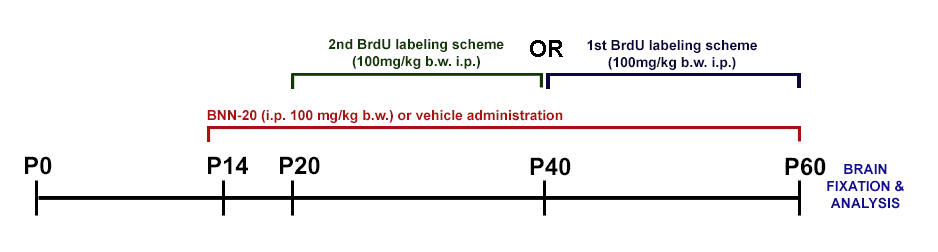

Supplement: Supplementary file 1 — Additional file 1. Graph depicting the administration schemes for BrdU and BNN-20, used for the in vivo labelling of the newborn TH+/BrdU+ neurons of the SNpc. [file 13287_2021_2398_MOESM1_ESM.tif]

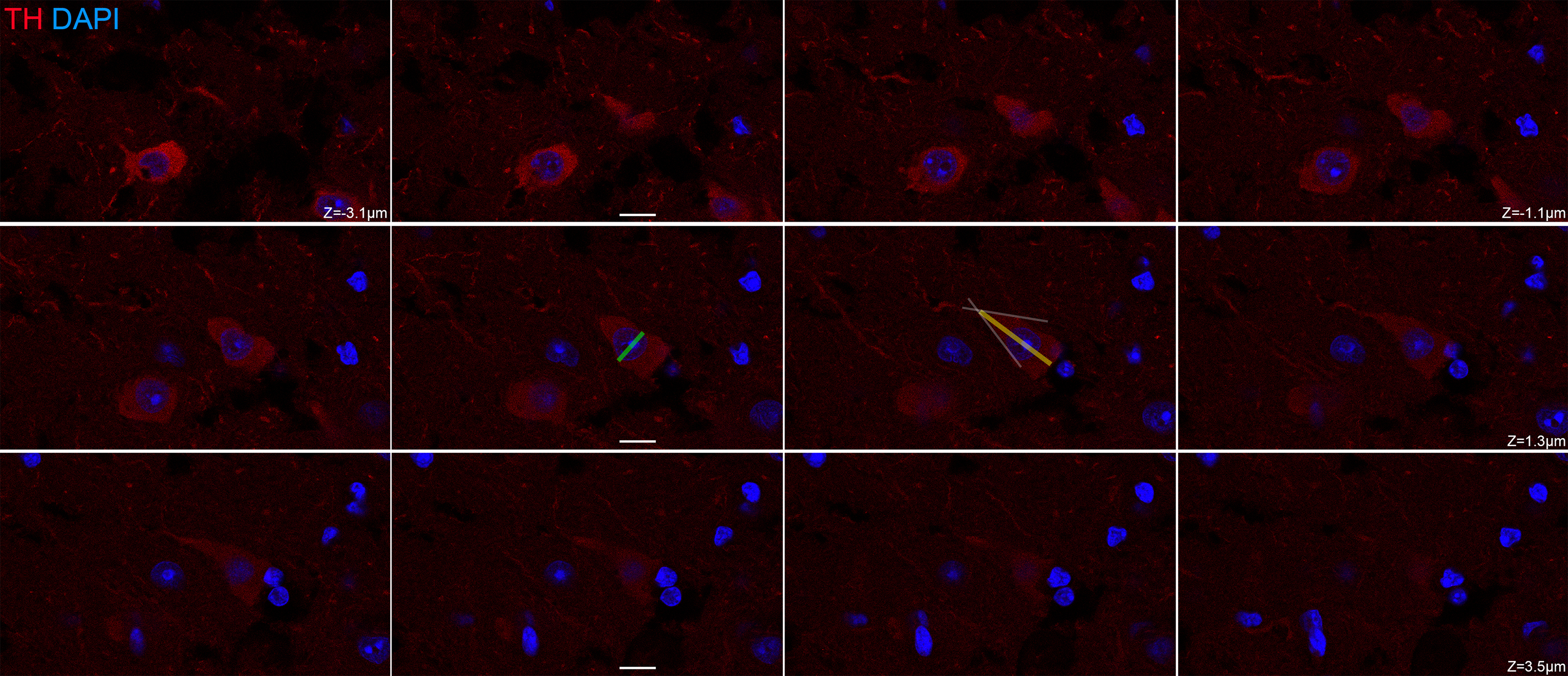

Supplement: Supplementary file 2 — Additional file 2. The method used in morphological analysis of TH+ neurons. Three dimensions of dopaminergic neurons were analyzed: the longest dimension of the cell soma (in yellow), the perpendicular to the longest (in green) and the shortest (defined by the depth – z, as indicated at the lowest right corner) [Scale bars = 10 μm]. [file 13287_2021_2398_MOESM2_ESM.tif]

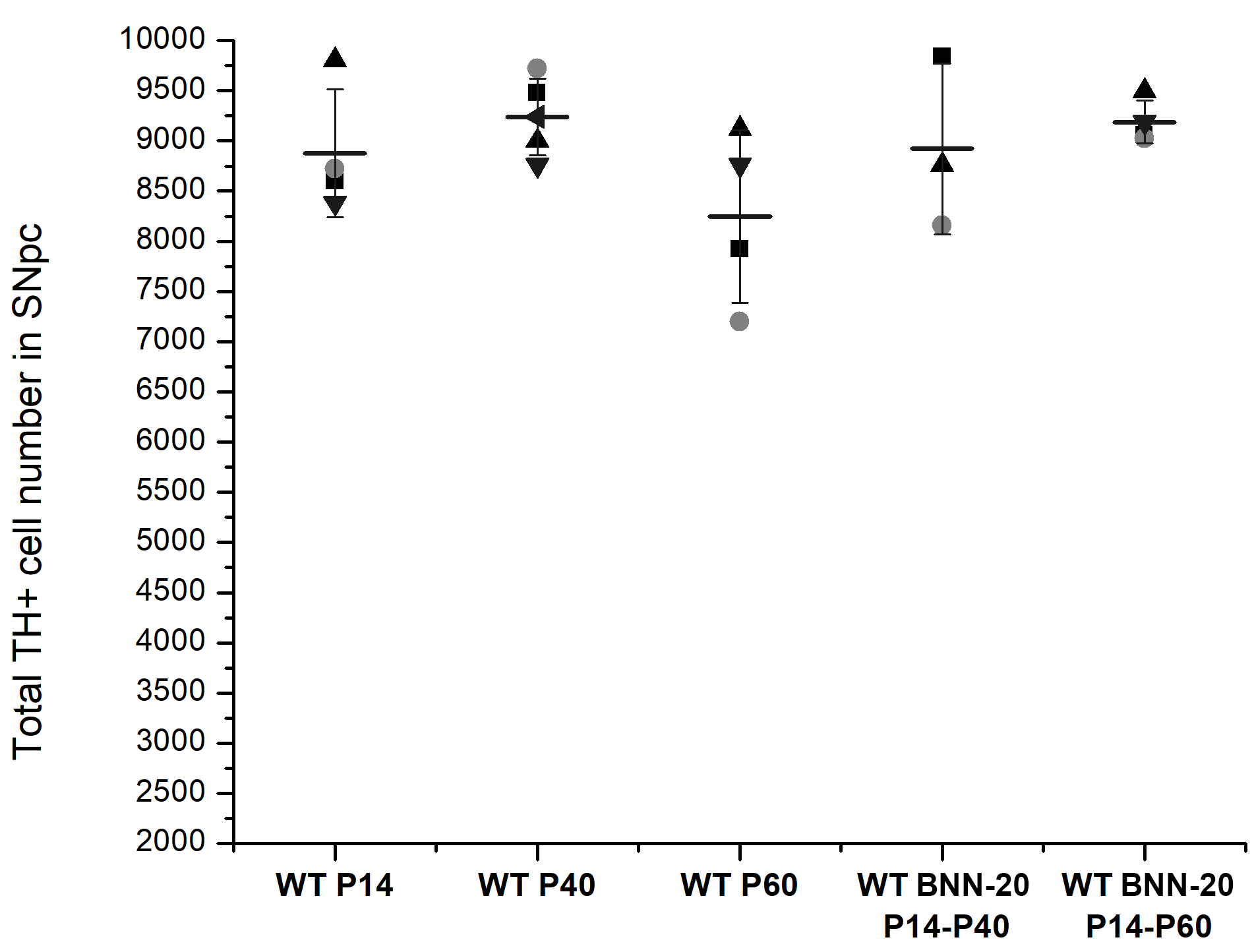

Supplement: Supplementary file 3 — Additional file 3. BNN-20 administration has no effect on the total dopaminergic neuron number of the WT SNpc. Total dopaminergic (TH +) cell number in the SNpc of wild-type (WT) mice, untreated (WT P14, WT P40, WT P60), or treated with BNN-20 from P14 to P40 (P14-P40) or to P60 (P14-P60) [n = 4 per group. Error bars are SDs; statistical analysis was performed using two-way ANOVA, followed by the Bonferroni post-hoc test]. [file 13287_2021_2398_MOESM3_ESM.tif]

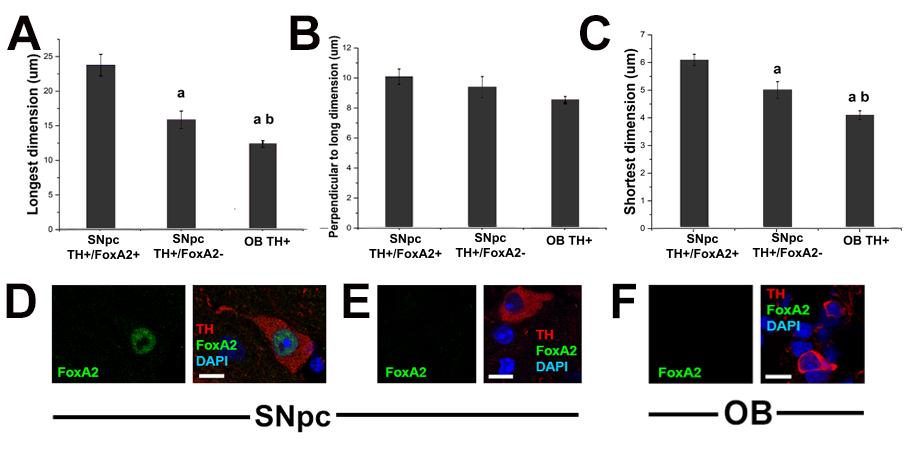

Supplement: Supplementary file 4 — Additional file 4. Morphology of different dopaminergic cell populations. Morphological analysis of the 3 dopaminergic neuron populations shown in Fig. 3A, by comparison of: (A) the longest dimension, (B) the perpendicular to the longest dimension and (C): the shortest dimension (depth) of the cell soma. Details are shown in Additional File 2 [In A: a: p < 0.001 compared to SNpc TH+/FoxA2+ neurons, b: p < 0.05 compared to SNpc TH+/FoxA2- neurons. In C: a: p < 0.01 compared to SNpc TH+/FoxA2+ neurons, b: p < 0.01 compared to SNpc TH+/FoxA2- neurons. Error bars are SEMs. n = 10 dopaminergic neurons per group. Statistical analysis was performed using one–way ANOVA (p = 0.000, F = 24.551 in E; p = 0.000, F = 18.849 in G), followed by LSD post hoc analysis.]. (D-E) Characteristic immunofluorescence images of (D) a TH+/FoxA2+ neuron of the SNpc, (E) a TH+/FoxA2- neuron of the SNpc and (F) a TH+/FoxA2- neuron of the OB [Scale bar = 10 μm]. [file 13287_2021_2398_MOESM4_ESM.tif]

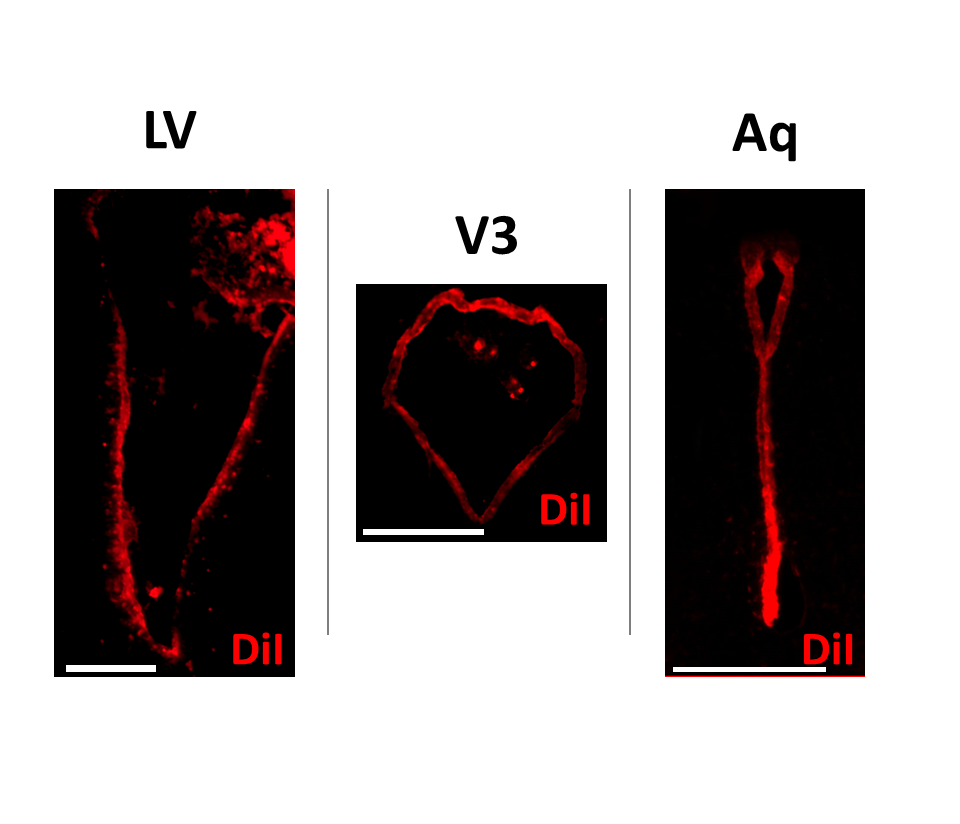

Supplement: Supplementary file 5 — Additional file 5. DiI staining of the lining of the ventricular systems. DiI incorporation in the ependymal and subependymal layers of the lateral ventricles (LV), the third ventricle (3 V) and the Aqueduct (Aq) after one unilateral DiI injection in the left LV [Scale bars = 500 μm]. [file 13287_2021_2398_MOESM5_ESM.tif]

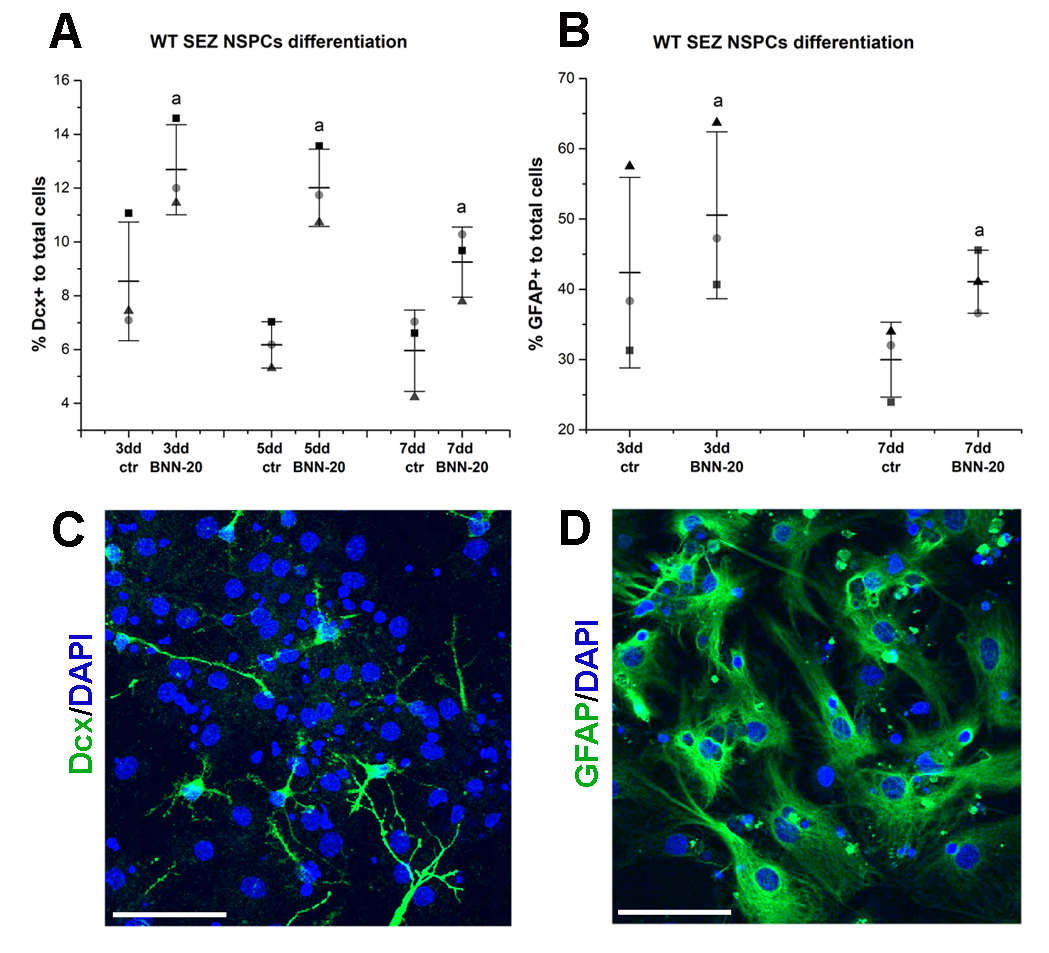

Supplement: Supplementary file 6 — Additional file 6. BNN-20 promotes neuronal and astroglial differentiation of NSCs in vitro (additional info). (A,B) Dot plots showing the percentages of (A) Dcx + immature neurons and of (B) GFAP+ (astrocytes) cells in cultures of wild-type (WT) SEZ-derived NSCs maintained in differentiation conditions for (A) 3, 5, 7 or (B) 3 and 7 days with or without (ctr) BNN-20 addition in the cell medium [Error bars are SDs. a: p < 0.05 using paired Student t-test analysis because the same cell samples were split in ctr or + BNN-20 conditions]. (C-D) Representative immunofluorescence images of cell cultures stained for Dcx (in C) and GFAP (in D) [Scale bars = 50 μm. n = 3 independent experiments]. [file 13287_2021_2398_MOESM6_ESM.tif]
